# Assessing Pandemic Response Implementation and the Workforce Impact Among Nurses and Midwives Internationally: Protocol for an Exploratory, Pragmatic, Online Descriptive Study

**DOI:** 10.2196/94179

**Published:** 2026-10-27

**Authors:** Allison Squires, Hillary J Dutton, Amal Mobarki, Simon Andrew Jones, Global Consortium of Nursing & Midwifery Studies, Raymond Aborigo

**Affiliations:** 1 Rory Meyers College of Nursing, New York University, 433 First Avenue, 6th Floor, New York, NY, 10010, United States, 1 2033147256; 2 School of Nursing, Rutgers, The State University of New Jersey, Newark, NJ, United States; 3 Center for Healthcare Delivery Science, Department of Population Health, Grossman School of Medicine, New York University, New York, NY, United States

**Keywords:** COVID-19, nurses, midwives, pandemic, mixed methods, mental health, low- and middle-income countries, global health, decolonizing research

## Abstract

**Background:**

The COVID-19 pandemic placed unprecedented demands on nurses and midwives worldwide, exacerbating existing workforce shortages and contributing to high rates of mental health issues and altered labor market dynamics. Nurses and midwives in low- and middle-income countries remain underrepresented in the literature.

**Objective:**

The aim of this study is to describe and compare the impact of pandemic response implementation across countries and contexts on the global nursing and midwifery workforce who worked and delivered care between March 2020 and December 2023.

**Methods:**

A pragmatic design guides this multinational, online, descriptive study. Eligible participants are nurses and midwives who cared for at least 1 patient with suspected or confirmed COVID-19. Country teams use opt-in convenience and network sampling, targeting 300 participants per country, or 60 participants for small island states. The self-administered 79-question instrument, hosted on Qualtrics XM software and available in 27 languages, was developed with country teams. It includes 8 open-ended free-text questions; 46 questions examining the impact of pandemic response implementation on mental health, career mobility, economics, and occupational exposures; and 25 demographic questions. After pilot-testing, data collection took place between July 2022 and June 2026. Summative content analysis combined with natural language processing techniques analyzed the qualitative data. Descriptive and bivariate analyses analyzed quantitative responses. Cross-country comparative analyses of quantitative data use funnel plots with binomial control limits to compare country responses and can accommodate unequal sample sizes. Psychometric evaluation of instruments developed and tested in this study is planned. Funding and ethics approval for the study were provided by the lead author’s university.

**Results:**

At the conclusion of data collection in June 2026, a total of more than 23,000 nurses and midwives from 108 countries engaged with this study’s online instrument, with over 14,000 completing more than 50% of all the questions. Descriptive analyses of partial samples have been published, as have the pilot tests of the methodology. The analyses specified here will use the complete dataset and are underway, with the first multinational analyses expected in 2027. Country teams will lead analyses specific to their country.

**Conclusions:**

Upon completion, this study will identify the common and unique factors that influenced pandemic response implementation relative to nurses and midwives during the COVID-19 pandemic, from low-resource to high-resource settings. Data may provide multilevel insights into the postpandemic workforce. Finally, this study will provide data that can inform country-level preparation to meet key articles of the World Health Organization Pandemic Agreement.

## Introduction

### Overview

The COVID-19 pandemic placed an extraordinary strain on health systems worldwide, and nurses and midwives, who constitute the largest proportion of the health workforce, have faced particularly severe challenges. With the passage of the World Health Organization (WHO) Pandemic Agreement in May 2025, ensuring that the nursing workforce is protected and sustained to respond to future pandemics is critical. The State of the World’s Nursing Report 2025 reported that in 2023, the global shortage of nursing personnel was estimated at 5.8 million, with projections indicating a decline to 4.1 million by 2030 [1]. An estimated 70% of this projected shortage will be concentrated in the African and Eastern Mediterranean regions [1].

COVID-19 intensified the shortage crisis, with severe impacts on working conditions, safety, and mental health for nurses and midwives worldwide [2-7]. For example, research indicates an extremely high prevalence of adverse mental health outcomes in health care workers during the pandemic, including anxiety, depression, insomnia, and stress [8]. A Canadian study of critical care nurses during COVID-19 shows high levels of psychological distress among participants, including symptoms of posttraumatic stress disorder, depression, anxiety, and stress [9]. In addition to psychological stress, nurses across countries and care contexts faced critical shortages of personal protective equipment (PPE), staffing deficits, and excessive workloads [10-14]. Therefore, these factors threaten both nurses’ well-being and the resilience of health systems. As the WHO has emphasized, investing in nurses and midwives is crucial to sustaining adequate health care and universal coverage for services [15].

The consequences for the nursing and midwifery workforce of the pandemic have outlasted its acute phase across country income levels. In the United States, although reported emotional exhaustion and workload moderated between 2022 and 2024, approximately 40% of nurses report an intention to leave nursing or retire within the next 5 years [16]. Some recovery in aggregate headcount has occurred in certain health care settings, so working conditions and intention to leave, rather than workforce size, may be driving these dynamics [17]. Internationally, health worker migration accelerated during the pandemic [18], and the International Council of Nurses reports a marked increase in nurse migration from low- and middle-income to high-income countries, driven largely by active recruitment by a small number of high-income states [19]. Because these effects fall unevenly on countries already short of nurses, establishing which conditions are common across health systems and which are specific to particular contexts is a prerequisite for workforce policy during the recovery period and for the next public health emergency.

Despite the need to understand the common and distinct structural differences between countries when implementing pandemic response protocols, there is limited comparative evidence on how the experiences of nurses and midwives vary across different countries and contexts. Furthermore, there are limited data specific to low- and middle-income countries (LMICs) and in emerging research environments where nurses and midwives are only beginning to lead research studies both about the workforces and the impact of the COVID-19 pandemic. Most existing studies are based on single-country or convenience samples, often from high-income settings, resulting in sparse global comparisons [20]. Voices from countries with underresourced research infrastructure have been underrepresented in the literature. In general, the COVID-19 nursing and midwifery workforce literature is weighted toward English-speaking countries, Western Europe, China, and Oceania, as well as settings with established health research infrastructure [3]. Even syntheses that pool large numbers of primary studies of health care workers’ mental health and burnout draw on an evidence base with this geographic skew [21,22]. The International Council of Nurses’ 2023 global report on the pandemic’s consequences for the nursing workforce drew only 10 of its 100 citations from low- and middle-income or emerging research–capacity contexts [23].

Three specific gaps in the literature highlight ongoing research deficits that could inform future pandemic preparedness. First, almost no study has collected occupationally specific mental health data on nurses and midwives using a common instrument across multiple countries simultaneously, which means cross-national comparisons currently require pooling studies that used different measures, time frames, and case definitions. Second, the organizational and resource conditions that shaped pandemic work, such as PPE availability, redeployment, and staffing adequacy, have rarely been measured alongside mental health and labor market outcomes in the same sample, so the relationships among them remain untested. Third, qualitative studies including nurses and midwives from LMICs do exist, but they are typically small and confined to a single country or setting [24-28]. Individually, they can describe the mechanisms operating in one health system; collectively, they cannot establish whether those mechanisms are shared across health systems or specific to particular ones, because they were not designed to be compared with one another.

In response, our international research collaboration—the Global Consortium of Nursing & Midwifery Studies (coordinated by our home institution and partners in Asia, Africa, the Americas, Europe, Oceania, and the Middle East)—launched a pragmatic, descriptive, multinational, online study of the impact of pandemic response implementation on nurses and midwives. This study seeks to explore and describe the experiences of nurses and midwives with the implementation of their country’s national pandemic response and its effects on their perspectives on pandemic-related working conditions, occupational safety, care practices, and personal health.

A multicountry descriptive design is required to answer these questions for 3 reasons. First, cross-national comparisons of the pandemic’s workforce effects are currently constrained by measurement heterogeneity. Meta-analyses of the mental health of nurses and health workers during the pandemic have repeatedly attributed substantial between-study heterogeneity to differences in the assessment scales and cutoff points used rather than to real differences between populations [29]. Others have found pooled estimates to vary significantly and have attributed this to the use of nonstandardized tools without established psychometric properties in the populations studied [30]. Neither of these studies noted the language of data collection, which may also have affected the findings, since standardized measures of common mental health issues are not widely available in lesser-spoken languages. A common instrument fielded simultaneously across many countries is therefore the only way to determine whether observed differences reflect real variation or measurement artifact. Second, structured items alone cannot explain why patterns differ between settings, since the organizational and cultural mechanisms behind them are not known in advance and cannot be prespecified as response options; narrative accounts are needed to identify them. Third, quantitative data alone cannot represent the experiences of nurses and midwives in settings where no prior workforce research exists, and qualitative data alone cannot establish how widely those experiences are shared. Collecting and merging both strands within a single instrument address each of these limitations.

### Study Aims and Research Questions

The primary aim of this study is to describe and compare (1) the experiences of nurses and midwives with pandemic response implementation in their countries and (2) the consequences of said implementation on them personally and professionally. We aim to include nurses and midwives across all countries within World Bank income-level classifications. The study has four research questions: (1) What are the common and distinct factors of pandemic response implementation practices that affect nurses and midwives in their place of work? (2) What organizational and resource conditions characterized their pandemic work and how are these similar and distinct across country income levels? (3) How do the personal consequences of pandemic response implementation (eg, mental health and coping strategies) vary by country and income group? (4) How does pandemic response implementation change labor market conditions for nurses, including migration and attrition?

A secondary objective of the study is to strengthen nursing and midwifery research capacity in participating countries through the collaborative authorship and data-sharing structure described in the Discussion section.

Ultimately, we expect the findings to elucidate key factors influencing nurses’ and midwives’ well-being and retention during a major global health crisis and to inform policy through policy briefs and scientific publications to better support this essential workforce. With the WHO’s approval of the Pandemic Agreement at the 2025 World Health Assembly, this study will provide valuable data to strengthen preparedness for future pandemics worldwide. Specifically, it will inform Articles 4, 6, 7, and 9 of the Pandemic Agreement, which address strengthening the health system and health workforce for pandemic preparedness, as well as improving research infrastructure [31-34].

### Background

This study involves country teams as equal partners, with local researchers leading recruitment and dissemination in each setting, and shared decision-making. The goal is to ensure that the voices of nurses and midwives, especially those from countries without robust research infrastructure, are captured and represented in the evidence. By developing research skills and ensuring equitable authorship across all participating countries, we aim to enhance nursing research capacity while generating actionable findings.

Participating countries were deliberately recruited across all 4 World Bank income strata. A goal of the analyses is to compare across those strata rather than describing any one of them. The emphasis on LMICs reflects a gap in the existing evidence rather than the scope of this study because the pandemic workforce literature is drawn overwhelmingly from high-income, English-speaking settings. Adding data from underrepresented countries is where this study makes its largest contribution, and comparison against well-studied high-income settings is what gives those data interpretive meaning. Including high-income countries is, therefore, not incidental to the design but necessary to it, since a study confined to low- and middle-income settings could describe conditions there but could not establish which features are specific to them and which are common to nursing and midwifery work under pandemic conditions everywhere.

### Instrument Development

#### Overview

Instrument development occurred between 2020 and 2021 and was first tested in English in the United States [10], followed by testing in multiple languages prior to expansion [35]. To summarize, data collection domains were identified in 3 steps. First, the initial English version was developed in the spring of 2020, drawing from anecdotal field reports about working conditions and the expertise of the principal investigator (PI) in the nursing workforce. It was then field-tested online in the United States, and the data were analyzed [10]. With the feasibility and methodology of the study established, when plans for international expansion developed in late 2020, the pilot testing teams were able to capitalize on a review of the emerging global literature to update and revise it. Revisions were informed by research on pandemic-driven factors influencing the implementation of nursing and midwifery roles. Refinements to the English version were also made to simplify the language to facilitate translation.

Given the urgency of the pandemic, a rapid consensus-making process was used to refine item wording, structure, and response formats through online, consensus-based content validation methods developed and refined by the study’s PI, which establish face validity and identify items that may require cultural adaptation [36-41]. The latter step is important because, with “health system language,” some concepts have no direct equivalents, such as job titles, cadre definitions, employment categories, and units of currency. The review process helped ensure that the appropriate local terms were used, even when they varied across countries that speak the same language. Country teams retain discretion to omit demographic items considered culturally inappropriate in their setting. These steps also helped ensure the instrument’s cross-cultural applicability in each country, as each team reviewed it. Finally, because there were no reliable and valid translations of mental health assessment instruments for all of the study’s languages, the team developed new measures as part of the project. For burnout measurement, the Mini-Z 3.0 [42] was revised and adapted for the registered and practical or vocational nursing roles. Psychometric assessments planned for the instrument are described in the Methods section.

#### Translation

Translation followed the systematic, multistep procedure for multicountry comparative health workforce studies that the PI previously developed and tested [41]. English served as the source language for all versions. A combination of machine translation and human validation facilitated translation for widely spoken languages (eg, Spanish, French, Arabic, Russian, and Mandarin), while traditional forward or back translation by humans was used for less widely spoken languages (eg, Georgian, Somali, and Khmer). The procedures described here establish equivalence at the point of translation; only invariance analyses can confirm it in the data. Since teams joined the study on a rolling basis, they implement the translation protocols prior to data collection.

#### Available Languages

The instrument is available in 27 languages: Arabic, Spanish, French, Chinese (simplified), Japanese, Urdu, Hebrew, Russian, Turkish, Swahili, Greek, Portuguese, Italian, Albanian, Slovenian, Finnish, Kazakh, Georgian, Mongolian, Khmer, Thai, Bahasa Indonesia, Latvian, Lithuanian, Polish, Estonian, and German. The full English master version of the instrument is provided in Multimedia Appendix 1, and the data dictionary is provided in Multimedia Appendix 2.

#### Response Formats

Unless otherwise noted, all participants see all items and have the option to not respond to any question or to answer “prefer not to say.” The proportion of participants responding to each prompt, overall and by country, will be reported alongside the qualitative findings so that readers can judge whose voices the narrative analysis represents.

Structured items capturing quantifiable data use 4 response formats. Dichotomous and small-category nominal items, each with a prefer-not-to-say option, are used for factual questions such as vaccination status, union membership, and confirmed infection. Ordered categorical frequency scales are used for the psychological symptom items, which are rated as “yes,” “sometimes,” “no,” “not sure,” or “prefer not to say,” and for the resource availability items, which are rated on a 5-point scale from 0=“not available” to 5=“always available.” Likert-type agreement and evaluative scales with 4 to 5 points are used for the Mini-Z items, covering agreement with statements about job satisfaction, values alignment, and job stress, as well as evaluative ratings of team efficiency, work-area atmosphere, workload control, and the overall work environment. Numerical open-response fields are used for age, planned retirement age, monthly salary in local currency, and counts of bereavements. Change-from-baseline matrices, rated as “more” or “better,” “about the same,” “less” or “worse,” “quit,” or “do not use,” are used for the self-care and substance use items, which ask participants to compare current with prepandemic behavior. Eight items collect qualitative data via free-text responses. The 5 conditional free-text boxes are displayed only to participants whose structured response makes them relevant, for example, those who report having experienced aggression from the public or having received extra pay. The response format is recorded for every item in the item-level data dictionary.

#### Data Content Domains

This section outlines the instrument’s content domains and the construct each domain measures. The demographics and employment domain describes the personal, professional, and employment characteristics of the respondent and the setting in which they practice. The organizational and resource factors domain measures the structural conditions of pandemic work, operationalized as organizational support, training provision, and the availability of essential clinical resources. The occupational exposures domain measures occupationally acquired risk and its institutional recognition. The mental health domain measures 2 distinct constructs: (1) burnout and the professional work environment, assessed with the validated Mini-Z, and (2) the frequency of self-reported psychological symptoms, differentiated between work and home. The coping and substance use domain measures change in self-care and substance use behaviors relative to the prepandemic period. The labor market and career outcomes domain measures employment security and disruption. The economic security, migration, and retirement intention domain measures income adequacy and intention to leave the post, the profession, the geographic setting, or the country. The vaccination domain measures vaccination status and perceptions of the national vaccination response. Finally, the care management and treatment observations domain captures nurses’ observations of clinical practice under pandemic conditions, treatment efficacy, and exposure to requests for non–evidence-based treatment. The free-text prompts capture the meaning participants attach to these experiences in their own words. A summary of the content domain details is provided in Table 1.

**Table 1.** Summary of data content domains.

Domains and measured constructs	Specific items or response formats
Demographics and employment	
Demographics	Country of residence and education, age, gender, heritage, qualification, specialty certification, years in practice
Work setting and context	Role type (frontline, educator, and administrator), clinical area, population served, employer type, organization type, university affiliation, geographic setting, union membership
Organizational and resource factors	
Employer support and training	Mental health support, training on PPE^a^, training on patient spiritual or emotional or psychological support
Resource availability	5-point scale (not available to always available) for evidence-based meds, PPE, IV^b^ equipment, oxygen equipment, mechanical ventilators
Operational changes	Redeployment and duration, extra pay or hazard pay, benefit or wage reductions, uncompensated prep or wrap-up time
Qualitative insights	Free-text: changes in practice, employer pandemic response, impacts on interprofessional teamwork
Occupational exposures	
Infection history and conditions	Confirmed COVID-19 infection, infection date, requirement to work while infected (including employer coercion)
Workplace policies and public interaction	Workplace-acquired infection classification, alternate housing offered, living away from home, public anger or aggression
Mental health	
Validated assessment	Mini-Z Burnout Assessment (10 items: job satisfaction, self-rated burnout, leadership alignment, efficiency, stress, documentation burden, atmosphere, workload control, work environment)
Self-reported symptoms	Work- and home-differentiated symptom sets capturing frequency of anxiety, depression, feeling overwhelmed, sadness, concentration difficulty, fatigue, irritability, sleep disturbance
Support and life impact	Professional help-seeking, provider type, barriers to help-seeking (cost, stigma, and access), bereavement (family or coworker deaths)
Qualitative insights	Free-text: main sources and temporal changes of work and personal stress
Coping and substance use	
Self-care strategies	Comparative ratings (more or better, same, less or worse) before versus during pandemic: exercise, eating, spiritual practices, relationship building, family or friend support, online communities
Substance use changes	Comparative frequency (more, same, less, quit, not applicable, prefer not to say): tobacco, alcohol, marijuana, injection drugs, narcotics, cocaine
Interpersonal impact	Impact on personal relationships with family, friends, or partners due to COVID-19 care
Labor market and career outcomes	
Employment patterns	Full-time, part-time, temporary, per-diem (and whether voluntary), holding multiple jobs
Disruption and recovery	Furlough or layoff history (March 2020-December 2023) and duration to re-employment inside or outside profession
Economic security, migration, and retirement	
Financial status	Monthly salary, salary sufficiency against poverty, primary wage earner status
Intentions and attrition	Intent to leave current post (next year), relocation plans (urban, suburban, rural, or international), intent to leave profession, planned retirement age
Vaccination	
Personal and workplace policy	Vaccination status, reasons for nonvaccination (free text), mandatory testing frequency
Public health context	Perceived population vaccination rate, rating of national overall vaccination effort
Care management and treatment observations	
Clinical practice and evidence	Frequency of requests from patients or families or physicians for non–evidence-based treatments in the preceding month
Qualitative insights	Free text: on-the-job training received, observed treatments that improved patient outcomes, observed treatments that failed to improve outcomes
Open-ended qualitative prompts	
Comprehensive narrative	Free-text prompts integrated across sections capturing stress sources, practice changes, employer response, teamwork, clinical observations, recommendations to improve pandemic response, and general additions

a PPE: personal protective equipment.

b IV: intravenous.

### Pilot Testing

Between 2021 and 2022, we conducted pilot studies in 13 countries and 8 languages, enrolling 2306 nurses and midwives [35]. These pilots provided invaluable experience that informed the final methodology for the wider study and produced the final instrument used for the study. From them, we developed the data dictionaries.

## Methods

### Study Design

This multinational, online, descriptive, multiple methods study is grounded in a pragmatic design. Pragmatic designs seek to generate results that can be quickly translated into real-world settings and inform policy [43,44]. We will collect data through an online questionnaire that combines structured items and free-text questions to generate both quantitative and qualitative data, as detailed in the Background section. While this study is not intended to follow mixed methods design specifications, it is possible that some data may converge; however, given the lessons from the pilot studies, the nature of the online data collection process, and the potential for wide variation between countries in terms of number of responses, we cannot guarantee sufficient sample sizes for each country to adhere to mixed methods protocols.

Therefore, reporting guidelines will be adhered to based on the type of data reported. For the quantitative data, the study will follow the STROBE (Strengthening the Reporting of Observational Studies in Epidemiology) guidelines for observational cross-sectional studies. For qualitative data, it will follow the SRQR (Standards for Reporting Qualitative Research) guidelines. Since the study collects data online, we will adhere to the CHERRIES (Checklist for Reporting Results of Internet E-Surveys) checklist reporting guidelines for both types of data. This study is also not registered because it is not a clinical trial.

### Ethical Considerations

Initial ethics approval has been obtained from the institutional review board of our home institution (institutional review board number FY2020-4440) and from ethics committees or administrative review boards in all participating countries, as required. Country partners had the option to accept the institutional review board approval. Amendments are filed in quarterly batches, as new countries join the study on a rolling basis. Any significant protocol amendments will be communicated to all sites and approved by the relevant boards. Before initiating the electronic survey, participants were presented with an informed consent in their preferred language. This consent clearly outlined the study’s objectives, the voluntary nature of participation, the specific expectations for respondents, and the potential risks and benefits. Participants agreed to participate in the study by selecting the “yes” option on the digital or paper consent form, indicating that they had read and understood the provided information and agreed to participate before proceeding with the survey. No personal identifiers were collected, IP addresses were anonymized, and a unique system-generated identifier was assigned to each respondent to track responses while ensuring anonymity. Participation was voluntary; country teams were allowed to offer incentives as resources, but most countries did not. All responses and data were securely stored in 2-factor–authenticated, password-protected digital environments, including the survey platform and electronic data management systems. Access to these data repositories was restricted exclusively to authorized members of the Global Consortium of Nursing and Midwifery Studies (GCNMS) research team to ensure the confidentiality and integrity of the dataset.

### Theoretical Framework

This study is grounded philosophically in pragmatism. Pragmatic approaches prioritize practical and achievable methods, recognize the contextual and situational nature of knowledge generation, and support the use of multiple data sources to characterize complex phenomena [43-46]. Pragmatism is also action-oriented, which aligns with the study’s goal of producing findings that can be translated rapidly into pandemic preparedness policy.

Because few established theories address pandemic response implementation from a health workforce perspective, the study also uses the structural determinants of health as a theoretical lens rather than a formal framework [47]. We posit that the structural factors shaping a health system’s pandemic response influenced how nurses and midwives experienced their work during the study period. This lens accommodates multilevel factors, from the individual to the national level, and permits the identification of determinants common across contexts as well as those specific to a single country.

### Study Population and Sampling

#### Participants

The study population consists of nurses (registered and practical or vocational) and midwives working in any practice setting who provided direct care to at least 1 patient with confirmed or suspected COVID-19 between March 2020 and December 2023.

Inclusion criteria included (1) registration or recognized qualification as a nurse or midwife, including registered nurses, licensed practical or enrolled nurses, midwives, and equivalent cadres such as auxiliary midwives as defined by each country’s health system; (2) having worked in any capacity, whether clinical, administrative, or educational, at any point between March 2020 and December 2023; (3) having provided direct care to at least 1 person with confirmed or suspected COVID-19 during that period; and (4) capacity to give informed consent and to complete the instrument in one of the study languages. Nurses and midwives currently employed, those who have left the profession but worked during the designated period, and those recently retired are all eligible if they meet these criteria.

Exclusion criteria are as follows: (1) students enrolled in entry-level nursing or midwifery education programs, (2) health workers other than nurses and midwives, (3) nurses and midwives who did not provide direct care to a person with confirmed or suspected COVID-19 during the study period, and (4) inability to complete the instrument in any of the study languages. No age restriction applies.

#### Sample Size

##### Overview

Because the study collects quantitative and qualitative data concurrently, the recruitment targets are justified separately for each strand. The target sample size for each country is 300 for large countries and 60 for small island states. The rationales for the sample size and its impact on each type of data are as follows.

##### Qualitative Strand

Because the qualitative data are collected through open-ended prompts embedded in the same instrument rather than through interviews, the qualitative sample is determined by the quantitative recruitment rather than sampled independently, and sequential assessment of saturation during data collection is not possible. The adequacy of the qualitative data will therefore be assessed after data collection closes, using the concept of information power: the number of narrative responses required depends on the specificity of the aim, the richness of the responses obtained, and the analytic strategy. For countries recruiting toward the 300 target, the resulting volume of free-text responses is expected to exceed the amount needed for thematic saturation in a conventional interview study. Notably, the 2021‐2022 round generated 6483 qualitative data points from 1814 respondents [35].

For small island states and countries with comparably small workforces, the target of approximately 60 was set on feasibility grounds. Some of these countries may only employ 100 nurses, for example. From the pilot study, the target of 60 yielded sufficient narrative data to characterize contextually specific experiences and to include the country in the cross-national comparison. Where the volume or richness of narrative data from a given country proves insufficient for country-level thematic analysis, that country’s responses will be analyzed only within the pooled cross-national corpus, and this will be reported.

##### Quantitative Strand

Our primary analytic goal for the quantifiable responses is descriptive and exploratory: to estimate frequencies of experiences and to compare patterns across countries. We have set a pragmatic target of at least 300 respondents per country, yielding binomial CIs of approximately ±10% around a 50% proportion (wider if the proportion is near 0% or 100%), and approximately ±10% for differences in proportions between country subgroups [48]. The target sample size will also provide samples large enough for the planned psychometric analyses of the newly developed measures in each language.

Power calculations based on the number of nurses in a country could not be performed across all countries due to known problems with data quality in international aggregates on the nursing workforce [1]. Furthermore, a power calculation is unnecessary for a descriptive and comparative design, and one could not be performed for every country, given the lack of uniform data on the global nursing and midwifery workforce. Nonetheless, for planned inferential analyses (eg, comparing mean burnout scores between regions), we note that a sample of 300 per group would have ~80% power to detect small-to-moderate effect sizes (Cohen *d*=0.35‐0.40) at α=.05. Because our analyses include both country-level and individual-level variables, we will also consider multilevel modeling if sample sizes justify.

### Sampling Approach

Because many participating countries lack complete data on the nursing workforce and have limited communication infrastructure, the study used a standardized sampling strategy based on voluntary participation. In practice, this meant using an “opt-in” approach, in which potential respondents could choose to participate in the study. This approach is consistent with recognized practices in survey research, where participants are recruited through nonprobability methods such as convenience and network sampling [49,50]. Country teams use a combination of convenience sampling and social network–based recruitment to engage participants, a strategy commonly used in large-scale health workforce studies during the COVID-19 pandemic [51,52].

### Participating Countries

Country teams are recruited through existing research partnerships and professional networks, with the explicit aim of representing countries across all 4 World Bank income classification strata (low-income, lower-middle-income, upper-middle-income, and high-income) and all 6 WHO regions. Each country team is led by a nurse or midwife. Where in-country research capacity within the nursing and midwifery professions is limited, the country lead is a partner experienced in conducting nonphysician health workforce research.

### Data Collection

The survey is administered online using the Qualtrics XM platform, a secure and General Data Protection Regulation–compliant system, and reporting follows the CHERRIES checklist [53]. On a limited basis, paper-based versions of the instrument are limited to settings where access to Wi-Fi services is inconsistent or requires participants to use phone minutes to complete the survey. Teams then enter data using an anonymous identifier into a Microsoft Excel spreadsheet that matches the online survey’s database, and the US team then uploads it to the Qualtrics system. Therefore, the US team does not see any of the paper-based surveys. Both data types are also collected simultaneously through the same self-administered instrument. There is no separate qualitative data collection exercise, and no interviews are conducted.

Participants are recruited principally through electronic channels, including direct links to the online survey and QR codes. To ensure broad reach, especially in low-resource settings, country teams use a multichannel approach that combines social media outreach on platforms such as Facebook, LinkedIn, and X (formerly Twitter) with messaging applications such as WhatsApp and Telegram, as well as posting to international nursing and midwifery listservs. Each country team completes at least 5 recruitment pushes during its recruitment period. QR codes and survey links are also shared in person at professional gatherings and institutional meetings.

Once consent is obtained after reviewing the form on the first page of the data collection system, the questionnaire incorporates adaptive questioning and conditional logic to display items relevant to each participant, reducing response burden and improving data quality. Questions are grouped across multiple screens, with a manageable number per page, to limit cognitive fatigue and maintain engagement. As stated previously, most questions include a prefer-not-to-answer or equivalent option, and no question is mandatory. Adaptive questioning and conditional logic restrict display to items relevant to each participant, reducing response burden and the associated risk of satisficing. Free-text fields are unlimited in length, and participants are explicitly encouraged to write at length. Participants complete the instrument once; there is no follow-up round and no repeated measurement. Median completion time and item-level completion rates are recorded by the platform and will be reported. Responses in all study languages are exported together and translated at the start of the qualitative analysis, as described in the Data Analysis Plan section. Participant recruitment, eligibility screening, data collection, and the planned analysis pathway are summarized in Figure 1.

**Figure 1.**
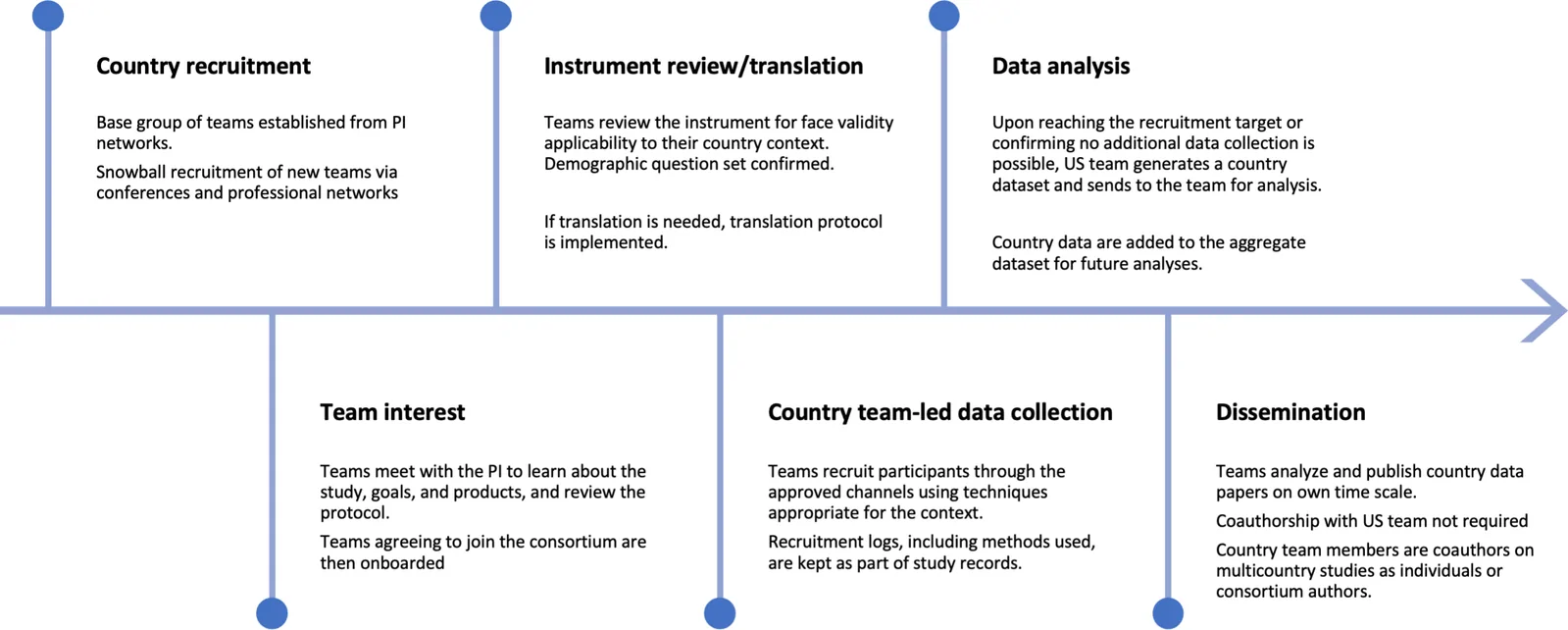
Country recruitment and implementation flowchart. Time to join the consortium project ranged from a week to several months, depending on the need for a data use agreement. PI: principal investigator.

Data collection began in July 2022. Teams joined the project through the end of 2025. Data collection closed in June 2026.

### Data Quality Assurance Measures

Several steps were taken to ensure that responses would generate the best-quality data possible. These included preventing duplicate submissions, conducting quality control checks, continuously monitoring, and implementing confidentiality measures.

#### Preventing Duplicate Submissions

The survey platform assigns a unique system-generated identifier to each respondent and monitors IP addresses, so responses originating from the same IP address are automatically flagged. Multiple bot-detection strategies available through the Qualtrics platform are enabled. Because logistical constraints in some countries require shared computer access, either because country team members enter responses on a single device or because several respondents complete the survey on the same device, flagged entries are not deleted automatically. Each is reviewed manually, and legitimate entries from shared devices are retained following verification, since the duplicate IP address reflects operational circumstances rather than fraudulent responding.

#### Data Quality Control

Entries flagged by the automated detection systems are reviewed weekly and manually by the project manager for completeness and internal consistency before inclusion in the analytical sample. Records are screened for implausible completion times and for straight-lining across matrix items. Country teams review their own country’s dataset for face-level plausibility against known workforce characteristics.

Since AI chatbots emerged in 2023 in the middle of data collection, there is a risk some participants have used them to answer questions. To date, our early triage of these responses has found that AI-generated qualitative responses are nearly identical to each other and are therefore eliminated during data cleaning procedures.

#### Data Monitoring

The core research team meets biweekly, and the full consortium meets quarterly to review recruitment progress against country targets, data quality indicators, and any reported adverse events. Country teams may meet with the PI at any time. Either body may recommend protocol modifications for methodological or safety reasons, which are then communicated to all sites and, where significant, submitted to the relevant ethics committees.

#### Data Confidentiality

No personal identifiers are collected, and IP addresses are anonymized in line with European Union standards for respondent deidentification. All responses are stored in password-protected digital environments with 2-factor authentication, comprising the survey platform and the electronic data management system, and access is restricted to 3 authorized members of the GCNMS research team. Qualitative responses are anonymized before analysis and reviewed only by trained team members. Narrative material will exclude any detail that could identify an individual participant or institution.

### Data Analysis Plan

#### Overview

Both types of data are used to answer the research questions, but the extent of each type’s contributions will depend on the quality of the final dataset. For all data types, data analysis will occur simultaneously. The US team leads the analysis of the entire dataset, while country teams lead analyses of their own datasets. For all qualitative analyses, teams follow the protocols outlined here. For quantitative analysis protocols, methods are adapted to the final country sample size with some commonalities. Analysis protocols for each data type and comparative country analyses are described in the following sections.

#### Qualitative Analysis

##### Overview

Free-text responses will be analyzed using summative content analysis combined with computational text mining in 6 sequential stages. Software used by teams will be specific to their local resources and may include (but is not limited to) MaxQDA v22, Taguette (open source), IRAMUTEQ, and/or ATLAS TI. All teams will follow a standard process to analyze their country’s data.

##### Stage 1: Corpus Preparation

Free-text responses will be exported from the survey platform, linked to the respondent’s anonymized unique identifier, and separated by prompt and by language. Responses in languages other than English will be machine-translated, with country team members proofreading the translations for accuracy against the source text.

##### Stage 2: Development of the Coding Framework

Two researchers per language will independently code a subset of responses to develop an initial coding framework of themes. The frameworks developed in each language will then be reconciled into a single framework for the pooled corpus. This coding framework is distinct from the item-level data dictionary and has not been finalized yet.

##### Stage 3: Coding

The full corpus will be coded against the agreed framework. Coding will be iterative, with coders refining and agreeing on categories as new material is encountered. The consensus-based open coding approach by Cascio et al [54] will harmonize coding across languages and countries. As there will be context- and language-specific codes, we will track both common codes and unique ones by country. This will serve as credibility and transferability checks on the analytic processes.

##### Stage 4: Summative Content Analysis

Summative content analysis counts the frequency of words as they appear in qualitative data [55]. It is a useful approach for analyzing open-ended, free-text responses captured through online data collection methods [56]. Our pilot work for this study found it to be highly complementary when using topic modeling approaches [10,35].

Therefore, for countries with more than 10 responses to a free-text question, the frequency of keywords and coded themes will be quantified across the corpus using summative content analysis. Their distribution will then be examined by country, income group, and WHO region. This is another step to ensure credibility and transferability of the analysis.

##### Stage 5: Topic Modeling

Latent Dirichlet allocation (LDA) will be applied as an automated technique to identify latent themes in the open-ended data [57-59]. It is used here for 3 reasons. First, given that the pilot test produced over 6000 qualitative data points, manual coding alone cannot satisfy at this scale. The corpus is expected to run to tens of thousands of responses across many languages, which exceeds what can be hand-coded consistently. Second, the LDA model derives structure from the text without reference to the coding framework. It assigns each response a continuous score on each topic, yielding a set of word clusters together with each response’s score on those clusters. Coherence measures and human interpretability will be used to select an appropriate number of topics, with each country team reviewing and evaluating reports based on their data. This stage runs in parallel with stages 3 and 4 and provides an independent check on the manually derived themes and provides a check on whether the manually developed themes are artifacts of coder expectation. In effect, it provides the dependability and confirmability checks on the data to ensure the trustworthiness of the findings.

##### Stage 6: Interpretation and Cross-National Comparison

Upon completion of the analyses, an aggregate thematic analysis of the entire dataset will be compared with the themes that emerged from the country-level analyses. Teams will determine where they observe core similarities and differences in the findings relative to their country to distinguish those that are common from those that are setting-specific. A descriptive matrix will be generated to highlight similarities and differences in theme names across countries. Reporting of the qualitative findings, when published, will follow the SRQR [60].

### Quantitative Analysis

#### Overview

All statistical analyses will be conducted in R (R Foundation for Statistical Computing) or the software available to the country team members. When not conducted in R, the code will be converted from the software used to R for study record-keeping.

#### Variables and Outcomes

Table 1 and the data dictionary denote the dependent variables of the study’s 3 outcome sets: self-reported mental health symptoms and burnout, measured as ordered categorical items and as the Mini-Z burnout categories; organizational and resource conditions, measured as ordered categorical availability and adequacy ratings and as binary indicators; and labor market outcomes, principally intention to leave the current post or the profession, measured as categorical items. The independent variables are participant-level characteristics (age, gender, profession, qualification, years in practice, role type, clinical area, employment status, union membership, and geographic setting) and country-level characteristics (World Bank income classification and WHO region). Age, planned retirement age, monthly salary, counts of bereavements, and duration of redeployment are numerical; all remaining variables are categorical, and most are ordinal. Where the organizational and resource conditions are examined as predictors of mental health or labor market outcomes, they function as independent variables; the role of each variable in each model will be stated explicitly when results are reported.

#### Descriptive Analyses

We will first perform descriptive analyses of all survey items. Continuous variables will be summarized by means (SDs) or medians (IQRs) as appropriate; categorical variables by counts and percentages. These summaries will be presented overall and stratified by country to highlight differences. Graphical displays (eg, bar charts for proportions and boxplots for scale scores) will aid interpretation.

#### Choice of Statistical Tests

Quantitative data analysis will use bivariate analyses (*χ*^2^ tests and *t* tests) and multivariable regression models to explore relationships between variables such as mental health outcomes and occupational context. Mixed-effects models will be used to account for clustering at the country level. Country-level subgroup analyses will also be conducted based on income classification and WHO region. Statistical significance will be assessed at the .05 level, but in the large descriptive context of this study, we will emphasize effect sizes and CIs over *P* values.

Because the data are drawn from many countries with differing sample sizes and response distributions, the distributional assumptions of each test will be checked before it is applied rather than assumed. Normality will be assessed by inspection of histograms and Q-Q plots, supported by formal tests when sample sizes permit, and homogeneity of variance will be assessed using the Levene test. Where the assumptions of parametric tests are met, independent-samples *t* tests and one-way ANOVA will be used for comparisons involving numerical outcomes; where they are not, the nonparametric equivalents (Mann-Whitney *U* and Kruskal-Wallis) will be used. Associations between categorical variables will be examined using *χ*^2^ tests of independence, with Fisher exact test substituted where expected cell counts are small. Ordinal outcomes will be analyzed using tests appropriate to their measurement level rather than being treated as continuous. Which test was applied, and why, will be reported for each analysis.

#### Analyses Within and Across Countries

For comparisons between countries, funnel plots will be used to present country-specific estimates relative to the overall pooled estimate. The overall effect will provide the center line, with control limits reflecting the expected variation around this estimate, based on the size of the country-specific sample. This approach explicitly accommodates unequal recruitment across countries, as estimates from countries with smaller sample sizes will have wider control limits, whereas those from larger countries will have narrower limits. Countries falling outside the control limits will be highlighted as potential outliers and investigated further. These comparisons will be considered exploratory and used to identify potential between-country heterogeneity rather than as formal hypothesis tests of differences between individual countries.

#### Multivariable Modeling

The primary comparative analysis will use funnel plots to present country-specific estimates in relation to the overall pooled estimate. The overall estimate will form the center line, with control limits reflecting the expected sampling variation according to each country’s sample size. This approach accommodates unequal recruitment across countries and will identify countries whose estimates differ from the overall estimate by more than would be expected from sampling variation alone. These countries will be highlighted for further investigation rather than interpreted automatically as statistically or clinically distinct.

Mixed-effects regression models will provide additional insight into between-country variation and adjusted associations. These models will include a random intercept for country to account for clustering of participants within countries and underlying heterogeneity between countries. This approach will allow the baseline outcome level to vary across countries while estimating overall adjusted associations. Where appropriate, between-country variation, including the intraclass correlation coefficient, will be reported. These analyses will complement rather than replace the primary funnel plot analyses.

#### Psychometric Evaluation of New and Existing Instruments

Because most items were developed for this study and have not previously been evaluated, psychometric analysis is a planned output of the study rather than an assumption. It will be conducted once data collection closes. As described in the Introduction section, content, semantic, and technical validation of the instrument’s items were determined by each country team prior to data collection.

For post–data collection psychometric validation of quantitative items, we will limit our analysis to exploratory factor analysis (EFA) to determine the item pool’s factor structure. EFA will use principal components analysis for factor extraction, and Varimax rotation will be applied to the factor solution for interpretability. Horn parallel analysis will be conducted to identify the optimal number of factors to retain, and the identified solution will be confirmed by multiple criteria, including examination of the screen plot, retained factors having eigenvalues >1 and having a minimum of 3 items with a primary loading (>0.4) on them, minimal cross-loadings, and simple structure. If the final sample size permits, the factor structure identified by the EFA will be validated using confirmatory factor analyses (CFAs). CFA model fit will be determined using multiple criteria (*χ*^2^, root mean square error of approximation, comparative fit index, and Tucker-Lewis index). Cronbach α will be calculated for subscales defined by identified factors to establish the instrument’s reliability. We will conduct these analyses across three levels: (1) the entire sample, (2) by language group, and (3) by country. These steps serve as additional checks on construct and criterion validity because regional linguistic variations within a single language may affect factor analysis results, and country context may alter them [61,62].

#### Missing Data

The extent and pattern of missing data will be described for each analysis variable, both overall and by country. Item nonresponse and explicit selection of a “prefer not to answer” response will be distinguished and reported separately where appropriate. Denominators will be reported for descriptive estimates to make the extent of available data transparent.

Some questionnaire items may not be administered in all countries because particular questions may be considered culturally, legally, or politically sensitive in specific settings. Such data will be treated as structurally missing and distinguished from participant-level nonresponse. The countries in which individual questions were not administered, and the reasons where applicable, will be documented. Analyses involving these variables will therefore be restricted to countries in which the relevant items were administered, and the number of countries and participants contributing to each analysis will be reported.

For items administered across countries, funnel plots will be used to examine country-level variation in missingness. Country-specific proportions of missing responses will be plotted against the relevant sample size, with the overall missingness rate as the center line and control limits reflecting expected sampling variation. This will allow countries with higher or lower levels of missingness than expected to be identified for further investigation, while appropriately accounting for differences in country-specific sample sizes.

For multivariable analyses, inclusion will be determined by the availability of the variables required for the specific analysis rather than by an arbitrary threshold for overall questionnaire completion. Complete case analysis will be used as the primary approach. The extent and patterns of missingness, and the associations between missingness and observed participant characteristics, will be examined to assess the plausibility of assumptions about the missing-data mechanism.

Where missing data are sufficiently extensive to potentially affect the findings, multiple imputation by chained equations will be considered as a sensitivity analysis, provided that the available data support a plausible imputation model. Structurally missing data arising because an item was not administered in a country will not be imputed. Results from imputed and complete-case analyses will be compared to assess the robustness of the findings. Variables with such extensive or systematically patterned missingness that reliable multivariable analysis is not considered appropriate will instead be reported descriptively, with the reasons for this decision documented.

Participants who consent but provide no substantive study data beyond the eligibility screen will be excluded from the analytical sample and accounted for in the participant flow. Otherwise, partially completed questionnaires will be included in descriptive analyses for items with available responses.

For qualitative analyses, absence of a response to a free-text question will not be interpreted as absence of the corresponding experience and will not be imputed. The number and proportion of participants who responded to each free-text prompt will be reported overall and, where appropriate, by country. Responses containing no interpretable substantive content will not be included in qualitative coding, and the number excluded for this reason will be reported.

## Results

Several key study milestones have been achieved. These are outlined in Table 2, with details discussed in succeeding paragraphs.

**Table 2.** Study timeline and accomplishments to date [2,10,35,63-67].

Phase	Period	Status	References
Instrument development, translation, and content validation	2020‐2022	Complete; reported as background	
Initial institutional review board approval	May 2020	Complete	
First multicountry pilot test	January 2021-February 2022	Complete; published	Squires et al 2026.
Country-level ethics approvals	2021-2026	Complete	
Data collection	July 2022-June 2026	Complete	
Recruitment of additional country teams	2022‐2025	Complete	
Data cleaning	July-December 2026	Underway	
Psychometric and multivariable analyses	September 2026-August 2027	Planned	
Qualitative analyses	April 2023-December 2027	Underway	
Quantitative analyses	April 2023-December 2027	Underway	
Country-specific publications and policy briefs	From 2027	Planned	
Multinational comparative publications	From 2027	Planned	

First, the instrument was successfully translated into 27 languages through rigorous validation processes, ensuring cultural relevance across diverse global settings. Languages include Arabic, Spanish, French, Chinese (simplified), Japanese, Urdu, Hebrew, Russian, Turkish, Swahili, Greek, Portuguese, Italian, Albanian, Slovenian, Finnish, Kazakh, Georgian, Mongolian, Khmer, Thai, Bahasa Indonesia, Latvian, Lithuanian, Polish, Estonian, and German. Psychometric evaluation has started.

Since July 2022, when data collection opened, teams have joined on a rolling basis, often from referrals from established partners. Upon joining, they are given at least 6 months to collect data. Countries with working teams as of July 2025 were Albania, Angola, Antigua and Barbuda, Argentina, Bangladesh, Bolivia, Brazil, Brunei, Cambodia, Cameroon, Chile, Colombia, Costa Rica, Côte d’Ivoire, Democratic Republic of Congo, Dominican Republic, Ecuador, Egypt, El Salvador, Estonia, Ethiopia, Finland, France, Georgia, Germany, Ghana, Greece, Grenada, Guatemala, Guyana, Honduras, Hong Kong/China, India, Indonesia, Iraq (Kurdistan), Italy, Jamaica, Japan, Kazakhstan, Kenya, Kosovo, Latvia, Lebanon, Lithuania, Malawi, Malta, Mexico, Mongolia, Montenegro, Nigeria, Pakistan, Panama, Paraguay, Peru, Philippines, Poland, Qatar, Rwanda, Saudi Arabia, Singapore, Slovenia, Somaliland/Somalia, South Africa, South Korea, Spain, St Lucia, Switzerland, Tanzania, Thailand, Trinidad and Tobago, Turkey, Uganda, United Arab Emirates, Uruguay, Venezuela, Zambia, and Zimbabwe (Multimedia Appendix 3).

In addition, because the study was distributed online and through networking, we sometimes obtained responses from countries not on our list of partner countries, as participants from these nations were able to respond because their languages were among the 27 translated versions of the instrument. As of July 2026, more than 23,000 nurses and midwives had accessed the study instrument, and more than 14,000 had completed at least 50% of the items, representing 108 countries.

In addition to the pilot studies, 2 consortium analyses have been published using preliminary data collected between 2022 and 2023. The first report presents descriptive findings on the self-reported mental health of nurses in 35 countries, drawn from participants who completed the survey between July 2022 and October 2023 [2]. Six country-level and regional analyses have also been published by individual teams [63-68]. None of the papers published to date include the comparative, multivariable, psychometric, qualitative, or integrated analyses specified in the Data Analysis Plan section, which require the complete dataset and could not be conducted until collection closed in June 2026.

With the conclusion of data collection in June 2026, data cleaning and preliminary descriptive analyses began in July 2026 and ran through December 2026; psychometric and multivariable analyses followed from January 2027, and qualitative and integrated analyses from mid-2027. Country-specific publications and policy briefs will follow country-level analyses, and the first multinational comparative analyses are expected in 2027. Policy briefs tailored to each participating country will be developed to support rapid translation of findings into practice.

## Discussion

### Principal Contributions

This multicountry study is among the most comprehensive efforts to date to document the experiences of nurses and midwives during the COVID-19 pandemic. It is one of the largest studies of nurses and midwives in LMICs ever conducted.

Its strengths lie in its broad scope, rigorous methods, and collaborative approach. By studying frontline workers across diverse global settings, including understudied LMICs, we aim to identify both common and context-specific challenges and resilience factors. The use of multiple data sources enriches our insights: qualitative narratives will provide depth and voice, while quantitative measures allow generalizability and hypothesis testing. The use of summative content analysis and topic modeling is innovative, enabling the quantification and integration of open-ended responses with structured survey outcomes.

### Methodological Innovation

Importantly, the study is grounded in decolonizing research principles that promote equity between investigators [69]. Scholars have increasingly called for global health research to center equity and local leadership [70]. This study responds to that call by ensuring that local investigators and results-led country teams are shared in participants’ languages and communities [71]. The structure of the GCNMS also supports research capacity development and more ethical collaboration across settings [72].

### Limitations

There are limitations of this study associated with its design, and the protocol includes specific measures to mitigate each, as detailed in the Methods section. To begin, convenience and opt-in sampling, combined with online-only administration, may skew participation toward digitally connected professionals and exclude those with limited internet access, particularly in LMICs [73]. Thus, the demographic profile of each country’s sample will be compared against available national workforce statistics (which may be limited, as previously mentioned) so that the direction and likely magnitude of any selection bias can be described rather than assumed. Because participants are not randomly selected, results will not be presented as nationally representative, but a comparison with available demographics may help determine this. To address these issues, results will be presented with CIs, and funnel plots will be used to illustrate cross-country uncertainty.

Asking participants to recall events over a multiyear period may introduce memory distortions; thus, recall bias is the most consequential threat to validity in this design and warrants fuller consideration. Since the reference period runs from March 2020 to December 2023, while recruitment ran from July 2022 to June 2026, the interval between the recalled events and the time of reporting ranged from roughly 2 to more than 6 years. It likely differs between participants recruited early and those recruited later in the study. Consequently, there are 3 potential data consequences. First, recall is uneven, with discrete, salient events, such as bereavement, redeployment, or furlough, which could be recalled more reliably over long intervals than diffuse states, such as symptom frequency or workload. Therefore, the symptom and workload estimates are likely to be less accurate than the event-based estimates and may be attenuated. Second, recall is reconstructive and colored by present circumstances. Participants who have since left the professions, for example, may recall the pandemic period more negatively than those whose circumstances recovered and stayed in them. Therefore, there is potential for bias whose direction cannot be assumed.

Third, because recruitment dates vary by country, differential recall intervals are confounded with country and its pandemic response period. An apparent cross-national difference, therefore, may partly reflect when a country’s team collected data rather than what its nurses and midwives experienced. Several measures address this. Items are phrased to cue reflection on the pandemic period as a whole rather than on specific dated incidents; the temporal reference period is stated explicitly in each relevant item; event-based items requiring counts are kept simple and bounded; and the survey completion date is recorded for every respondent, so recall interval can be modeled as a covariate and its association with the outcomes tested directly rather than assumed absent. Where the recall interval is associated with an outcome, that association will be reported, and the affected estimates will be interpreted with corresponding caution. For symptom reports, prevalence estimates will be reported with CIs and displayed on funnel plots so that between-country differences are interpreted in light of sample size rather than at face value. The narrative responses also provide a partial check, since accounts that remain internally detailed and specific over long intervals lend more confidence than accounts that have flattened into generalities.

In addition, while translations were carefully validated, subtle cultural or linguistic differences may affect how items are understood [74]. Mental health data are self-reported, which introduces subjectivity and potential under- or overreporting of symptoms; therefore, we report frequencies rather than diagnostic prevalence across measures [75]. Finally, social desirability may affect responses to sensitive items on substance use and intention to leave. Anonymity, the absence of any personal identifiers, and a prefer-not-to-answer option on every item are intended to reduce this effect, and nonresponse rates for those items will be reported alongside the substantive results.

### Policy Implications

The study’s policy implications follow directly from its 4 research questions and the domains they cover and are framed to apply across health systems at all income levels rather than to any single country. On mental health and burnout, cross-national estimates will indicate where employer-provided mental health support is absent and to what extent, informing the workforce mental health provisions of Article 7 of the Pandemic Agreement. On organizational and resource conditions, comparative data on PPE and essential resource availability, redeployment, and training received will identify which structural protections were actually delivered to frontline staff and which failed, informing national stockpiling, surge staffing, and training standards. On labor market outcomes, estimates of intention to leave the post or the profession, together with data on economic security and migration intention, will indicate where pandemic working conditions translated into workforce loss and in which direction that loss flowed, which bears directly on retention policy and on the ethics of international recruitment from countries already experiencing shortage. The narrative data will explain why these patterns differ across settings, which makes the findings transferable rather than merely comparative: a policy that succeeded in one health system can be assessed against the conditions that made it work. Tailored 2-page policy briefs in each partner country’s language, and a public data dashboard, are planned so that findings reach national decision-makers rather than remaining in the academic literature.

### Future Directions

In light of the WHO’s approval of the Pandemic Agreement at the 2025 World Health Assembly, the insights generated by this research offer timely, relevant data to support efforts to strengthen health systems and enhance global preparedness for future pandemics. Findings have the potential to substantively inform Articles 4, 6, 7, and 9 [31].

### Conclusions

This protocol describes a study designed to answer 4 specific questions about the impact of pandemic response implementation on nurses and midwives who worked through the COVID-19 pandemic, across countries at all income levels. On completion, the study will have several products. First, this study will provide comparative estimates of mental health symptoms, burnout, and pandemic-related loss, with country-level variation made visible rather than averaged away. Second, this study will provide a description of the organizational and resource conditions under which this care was delivered during the COVID-19 pandemic, which can inform future pandemic preparedness efforts. Finally, this study will help improve understanding of the intention of nurses and midwives to leave posts and professions, together with the economic and migration pressures behind this intention. Two further contributions follow from the design itself: a set of psychometrically evaluated measures of worker experience with pandemic response implementation, available in multiple languages for reuse, and evidence generated by research teams in countries where nursing and midwifery research capacity is still being built. Together, these outputs address a documented gap in the global evidence and provide a basis for the health workforce provisions of pandemic preparedness planning under Articles 4, 6, 7, and 9 of the WHO Pandemic Agreement.

## Supplementary material

10.2196/94179 Multimedia Appendix 1 Study instrument.

10.2196/94179 Multimedia Appendix 2 Data dictionary.

10.2196/94179 Multimedia Appendix 3 List of consortium countries by data collection round as of 2025.

## Abbreviations

CFA: confirmatory factor analysis
CHERRIES: Checklist for Reporting Results of Internet E-Surveys
EFA: exploratory factor analysis
GCNMS: Global Collaborative on Nurses and Midwives Study
LDA: latent Dirichlet allocation
LMICs: low- and middle-income countries
PI: principal investigator
PPE: personal protective equipment
SRQR: Standards for Reporting Qualitative Research
STROBE: Strengthening the Reporting of Observational Studies in Epidemiology
WHO: World Health Organization
