# Supplementary material for: Assessing Pandemic Response Implementation and the Workforce Impact Among Nurses and Midwives Internationally: Protocol for an Exploratory, Pragmatic, Online Descriptive Study
Source: JMIR Res Protoc. 2026 Oct 27;15:e94179. doi: 10.2196/94179 (PMC13712254; doi:10.2196/94179)
Supplement: Supplementary file 1 [file resprot-v15-e94179-s001.pdf]

Global COVID Nursing & Midwifery Study

Informed Consent Section

NYU Rory Meyers College of Nursing Informed Consent

Title of the Research Study: Global Nursing & Midwifery Perspectives of Working During the COVID-19 Pandemic

Principal Investigator: (name, address, phone and email):
Allison Squires, PhD, RN, FAAN
433 First Avenue
New York, NY 10010
(212) 992-7074
aps6@nyu.edu

Emergency Contact: (name, address, phone and email):
Allison Squires, PhD, RN, FAAN
433 First Avenue
New York, NY 10010
(212) 992-7074
aps6@nyu.edu

Please read the following to the participant or have them read the informed consent section.

You are being asked to take part in this research study because you may provide care to patients that have COVID-19. Your participation is voluntary, which means you can choose whether or not to participate. Before you make a decision, you will need to know the purpose of the study, the possible risks and benefits of being in the study and what you will have to do if you decide to participate.

- The purpose of the study is to learn more about nurses' and midwives' experiences living and working during the COVID-19 pandemic.
- You are being asked to join this study because you may have provided care and treatment to people infected with COVID-19 during the last two years.
- Nurses and midwives across the world will share their experiences about providing clinical management to patients with COVID-19 and subsequent patient outcomes.
- Data collection for this study will continue until June 30, 2026.
- There is just the one survey.
- During the survey you will be asked to share your experiences related to your clinical practice, managing COVID-19 patients, the resources/treatments you want or think would be helpful for your patients but have not been provided or received, the outcomes you think are most important to your patients and your clinical management. We will also ask you about how your experiences have affected you personally.
- One risk is that you might be asked questions that you might be sensitive to or make you slightly embarrassed to answer, however, once again your participation in the survey is completely voluntary and anonymous. We ask that you answer questions to the point that you feel comfortable in sharing. You will be asked to respond to all questions in the survey.
- There is no benefit to you. However, your participation could help us understand how to improve health care delivery during a pandemic, how to improve support for nurses and midwives working during a pandemic, and the need for changes in policy, which can benefit you indirectly. In the future, this may help patients, nurses, and midwives experience better outcomes during a pandemic.
- Participating in this study is voluntary. You can withdraw from the study at any time.
- There are no costs to you for participating.
- Research team members will have access to your survey responses. All information will be deidentified to maintain your anonymity.
- Information from this study that is published or presented at scientific meetings will not contain your name.
- We will create a research identification number that will be kept in a secure, password protected computer file. This number will be used instead of your name on all records. Only the research team members will have access to this list of codes. The Institutional Review Board (IRB) at the New York University and if applicable, your home country will have access to these records if required.

Your participation in this research study is voluntary. If you decide not to participate, you are free to decline to participate.

If you have questions about your participation in this research study or about your rights as a research subject, make sure to discuss them with the study investigator or members of the study team. You may also call the Office of Regulatory Affairs at the New York University at (212) 998-4808 to talk about your rights as a research subject.

2. Do you agree to participate in the study? Please check a box.

- □ Yes (1)
- □ No (2)

3. What is your profession? Even if you are trained as both a nurse and midwife, please choose one.

- □ Nursing (1)
- □ Midwifery (2)

We will now ask you a series of questions about yourself, your work, and what it has been like living and working during the COVID-19 pandemic.

Some questions will ask you to write out your response. Your words and how you describe your experiences are very important. Please write as much as you can!

Let's begin with a few questions about where you are living and working.

4. What country do you live and work in? (Q5)

____

In what country did you receive your nursing or midwifery education? (Q6)

____

5. What is your age? (Q7) ______

6. What is your heritage? (Q9)

- □ Asian or Pacific Islander (1)
- □ African origin (2)
- □ Latin American (3)
- □ Caribbean (4)
- □ European (5)
- □ Indigenous origins (6)
- □ Middle Eastern (7)
- □ Mixed heritage (8)
- □ Other (9): ____
- □ Prefer not to respond (10)

7. What is your gender? (Q10)

- ○ Female (1)
- ○ Male (2)
- ○ Other (3): ________________

SECTION ON MENTAL HEALTH

This series of questions focuses on any stress you may have experienced during the last few years when living and working during the pandemic.

8. Please describe your main sources of <u>work-related stress</u> during the pandemic. If your sources of work-related stress have changed over time, please describe how. Take as much space as you need. **(Q59)**

9. Please describe your main sources of <u>personal stress</u> during the pandemic. If your sources of personal stress have changed over time, please describe how. Take as much space as you need. **[NQ1]**

To answer the question, please check one (1) box.

10. Did (does) your employer provide appropriate mental health support services for employees? **(Q37)**

- ▢ Yes (1)
- ▢ Sort of (2)
- ▢ No (3)
- ▢ Not sure (4)
- ▢ Prefer not to say (5)

11. During the last two years, as a nurse or midwife, have you experienced any anger or aggression from the public? **(Q43)**

- ▢ Yes. Please describe in the space below. (1)

  ____

  ____

  ____
- ▢ No (2)
- ▢ Not sure (3)
- ▢ Prefer not to say (4)

12. Have you experienced any of the following symptoms <u>at work</u> on a regular basis since the pandemic began (Check all that apply): **(Q48)**

Symptom	Yes	Sometimes	No	Not Sure	Prefer Not to Say
Anxiety					
Depression					
Feeling overwhelmed					
Feeling sad frequently					
Difficulty concentrating					
Feeling tired most days					
Feeling irritable most days					
Hard to concentrate					

13. Have you regularly experienced any of the following symptoms <u>after work</u> when at home (Check all that apply): **(NQ10)**

Symptom	Yes	Sometimes	No	Not Sure	Prefer Not to Say
Anxiety					
Depression					
Feeling overwhelmed					
Feeling sad frequently					
Difficulty concentrating					
Feeling tired most days					
Feeling irritable most days					
Problems sleeping or staying asleep					

14. Have you sought professional help for the symptoms? (Q49)

- ◻ Yes (1)
- ◻ No (2)
- ◻ Prefer not to say (3)

If you answer YES to the question above, please go to question 15.

If you answer NO to the question above, please go to question 16.

If you answer "Prefer not to say" to the question above, please go to question 17.

15. Since you have sought help for your symptoms related to anxiety, depression, or being overwhelmed, from whom do you or did you receive professional help? (Q50)

- ◻ Workplace counselor (1)
- ◻ Psychologist (2)
- ◻ Social worker (3)
- ◻ Pastoral care (4)
- ◻ Psychiatric mental health nurse practitioner (5)
- ◻ Psychiatrist (6)
- ◻ Other (7) ____

16. If you have not sought help for your symptoms related to anxiety, depression, or being overwhelmed, why not? Please check all that apply. **(Q51)**

- ▢ There are no mental health services near where I live or work. (1)
- ▢ I don't know where to start looking. (2)
- ▢ My health insurance does not cover mental health counseling. (3)
- ▢ It is too expensive for me right now. (4)
- ▢ Support from my coworkers, friends, or family is enough for now. (5)
- ▢ I am afraid of being stigmatized. (6)
- ▢ I cannot get an appointment, in-person or virtual, because no one is available.
- ▢ Other (7) ____

17. Have your personal relationships (such as those with family members, friends or partners) been affected , because of your work with people infected with COVID-19? **(Q52)**

- o Yes. How? Please describe. (1)
  ____
  ____
- o Somewhat. How? Please describe. (2)
  ____
  ____
- o No (3)
- o Prefer not to say (4)

Questions 18 and 19 may be culturally sensitive. Please ask them of participants according to your cultural norms.

18.a. Many people report different self-care strategies for getting through the pandemic. Compared to what you did before the pandemic, what are you doing now to care for yourself? **(NQ12.a)**

Strategy	Doing More or Better	About the Same	Doing Less or Worse	Prefer Not to Say	Do Not Use
Exercise					
Eating habits					
Spiritual practices					
Building new, supportive relationships					
Talking with family					
Talking with friends					
Support from online communities					

18.b. Many people report other coping strategies for getting through the pandemic. Compared to what you did before the pandemic, what are you doing now to cope? **(NQ12.b)**

Strategy	More	About the Same	Less	Quit	Not Applicable /Do Not Use	Prefer Not to Say
Smoking tobacco						
Drinking alcohol						
Smoking marijuana						
Injection drug use						
Narcotics use						
Cocaine use						

19. What is your sexual orientation? **(Q11)**

- Heterosexual (1)
- Homosexual (2)
- Bisexual (3)
- Other (4)
- Prefer not to say (5)

20. Did any family members or relatives die of COVID-19? **(NQ15)**

- ☐ Yes (1)
  - How many? ______
- ☐ No (2)

21. Did you have any friends die of COVID-19? **(NQ16)**

- ☐ Yes (1)
  - How many? ______
- ☐ No (2)

22. Did you have any co-workers die of COVID-19? **(NQ17)**

- ☐ Yes (1)
  - How many? ______
- ☐ No (2)

< GO ON TO THE NEXT PAGE>

SECTION ON THE WORKPLACE & PANDEMIC RESPONSE

Now we would like to know about where you have been working during the pandemic. Please answer the questions based on your current job.

23. Please describe how your practice as a nurse or midwife has changed during the pandemic. **(Q54)**

24. Please describe how your employer has responded to the pandemic. What did they do well? What did they not do well? **(Q55)**

25. Please tell us about how the pandemic affected teamwork in your workplace. Please share about teamwork with fellow nurses or midwives, physicians, and other allied health professionals. **(Q56)**

DEMOGRAPHIC QUESTIONS

26. How many years have you worked as a nurse or midwife? **(Q12)**

- ◻ 0-3 years (1)
- ◻ 4-6 years (2)
- ◻ 7-10 years (3)
- ◻ 11-15 years (4)
- ◻ 16-20 years (5)
- ◻ 21-25 years (6)
- ◻ 26+ years (7)
- ◻ Prefer not to say (8)

27. At what age do you plan to retire? **(NQ13)** _____

28. What population is the focus of your work? You may select more than one answer. **(Q13)**

- ◻ Mothers and/or children under 5 years of age (1)
- ◻ Children ages 6 to 18 years of age. (2)
- ◻ Adults between 19 and 64 years of age. (3)
- ◻ Older adults aged 65 years or more. (4)

29. Which option best describes your role in nursing or midwifery? **(NQ12)**

- ◻ Practitioner or frontline worker
- ◻ Educator
- ◻ Management/Administration
- ◻ Other:____

30. What is your area of clinical practice? Choose the closest match. **(Q14)**

- ◻ Intensive care unit/ward (1)
- ◻ Intermediate or step-down unit/ward (2)
- ◻ Emergency department (3)
- ◻ Medical-surgical combination: Please specify below. (4)
  ____
- ◻ General Medicine (5)
- ◻ General Surgery (6)
- ◻ Labor & Delivery/Obstetrics/Maternal-Child Health (7)
- ◻ Psychiatric & mental health nursing (8)
- ◻ Operating Room/Theatre (9)
- ◻ Long term care, physical rehabilitation, or nursing home (10)
- ◻ Public Health/Community Health/Health Promotion (13)
- ◻ Other (11) ____
- ◻ Prefer not to say (12)

31. What is the highest nursing or midwifery degree you hold? **(Q15)**

- ◻ Technical, enrolled, diploma, certificate, or associate's degree. (1)
- ◻ Bachelor's degree (2)
- ◻ Master's degree (3)
- ◻ Doctorate (4)
- ◻ Prefer not to say. (5)

32. Do you have a specialty certification? **(Q16)**

- ◻ Yes (1)
- ◻ No (2)

33. What type of organization do you work for? **(Q17)**

- ◻ Primary or community-based care (1)
- ◻ Ambulatory/Outpatient care (2)
- ◻ Hospital (3)
- ◻ Home health care (4)
- ◻ Staffing agency (7)
- ◻ Other (5) ____
- ◻ Prefer not to say (6)

34. Is your organization affiliated with a university or school of nursing? **(Q18)**

- ◻ Yes (1)
- ◻ No (2)
- ◻ Not sure (3)
- ◻ Prefer not to say (4)

35. Where is your place of work? **(Q19)**

- ◻ Urban setting (1)
- ◻ Suburban setting (2)
- ◻ Rural setting (3)
- ◻ Prefer not to say (4)

36. What kind of organization is your employer? **(NQ15)**

- ◻ Public (1)
- ◻ Not for Profit (2)
- ◻ Non-governmental organization (3)
- ◻ Private (4)
- ◻ Other (5)
- ◻ Prefer not to say (6)

37. Are you a member of a labor union? **(NQ20)**

- ◻ Yes (1)
- ◻ No (2)

38. What is your employment status? **(NQ6)**

- ◻ Full-time (1)
- ◻ Part-time (<24h/week) (2)
- ◻ Temporary contract (3)
- ◻ Per-diem (4)
- ◻ Other (5): ____

If you answered "Per Diem or Temporary Contract, please answer Question 38.a..

38.a. Since you are on a temporary or per-diem contract, is that: **(NQ7)**

- ◻ By choice (1)
- ◻ Your only option (2)
- ◻ Other (3): ____

39. Do you have more than one job in nursing or midwifery right now? **(NQ8)**

- ◻ Yes (1)
- ◻ No (2)

40. Do you have another job outside of nursing or midwifery right now? **(NQ9)**

- ◻ Yes (1)
- ◻ No (2)

41. At any point between March 2020 and the present, were you furloughed or laid off from a nursing or midwifery job? **(Q40)**

- ◻ Yes (1)
- ◻ No (2)

If you answered NO to question 41, please go to Question 42.

If you answered "YES" to question 41, please answer the following questions by checking one box per row.

41.a. When you were furloughed or laid off from your nursing or midwifery job, how long did it take you to:

	1 week to 3 months	4-6 months	7+ months	Not applicable
Return to your original job (1)				
Find temporary employment as a nurse or midwife (2)				
Find temporary employment outside of nursing or midwifery (3)				
Find part-time or full-time employment in nursing or midwifery (4)				
Find part-time or full-time employment outside of nursing or midwifery (5)				

< GO ON TO THE NEXT PAGE>

MINI-Z BURNOUT ASSESSMENT

Please answer each question by checking the box that best represents your experiences.

42. Overall, I am satisfied with my current job. (NQ3.1)

- □ Strongly agree (1)
- □ Agree (2)
- □ Disagree (3)
- □ Strongly disagree (4)

43. Using your own definition of "burnout", please choose one of the answers below. (NQ3.2)

- □ I enjoy my work. I have no symptoms of burnout. (1)
- □ I am under stress, and don't always have as much energy as I did, but I don't feel burned out. (2)
- □ I am definitely burning out and have one or more symptoms of burnout, e.g. emotional exhaustion. (3)
- □ The symptoms of burnout that I'm experiencing won't go away. I think about work frustrations a lot. (4)
- □ I feel completely burned out. I am at the point where I may need to seek help. (5)

44. My professional values are well aligned with those of my department leaders. (NQ3.3)

- □ Strongly agree (1)
- □ Agree (2)
- □ Disagree (3)
- □ Strongly disagree (4)

45. The degree to which my care team works efficiently together is: (NQ3.4)

- □ Excellent (1)
- □ Good (2)
- □ Satisfactory (3)
- □ Low/needs improvement (4)
- □ Poor (5)

46. I feel a great deal of stress because of my job. (NQ3.5)

- □ Strongly agree (1)
- □ Agree (2)
- □ Disagree (3)
- □ Strongly disagree (4)

47. I stay after my shift is done to finish documentation (paper or electronic): (NQ3.6)

- ◻ Every time I work (1)
- ◻ 2-3 times/week (2)
- ◻ Once per week (3)
- ◻ Once every two weeks (4)
- ◻ Once a month (5)
- ◻ Very rarely (6)

48. If I have to stay after my shift ends to finish documentation, I get paid overtime. (NQ3.7)

- ◻ Yes (1)
- ◻ Sometimes (2)
- ◻ No (3)

49. Which number best describes the atmosphere in your primary work area? (NQ3.8)

- ◻ Calm (1)
- ◻ Mostly quiet with periods of busyness (2)
- ◻ Busy, but reasonable (3)
- ◻ Constant activity, occasionally very intense (4)
- ◻ Hectic, chaotic (5)

50. My control over my workload is: (NQ3.9)

- ◻ Excellent (1)
- ◻ Good (2)
- ◻ Satisfactory (3)
- ◻ Low/needs improvement (4)
- ◻ Poor (5)

51. Overall, my work environment is: (NQ3.10)

- ◻ Excellent
- ◻ Very good
- ◻ Good
- ◻ Not too good
- ◻ Bad

Economics

52. From your primary job, what is your monthly salary? ____________________
Please respond in your country's currency.

53. Is your monthly salary enough to keep you out of poverty? **YES** **NO**

54.Is your monthly salary enough to keep your household out of poverty? **YES** **NO**

55. Are you the primary wage earner in your household? **YES** **NO**

Migration/Attrition Assessment

56. Are you thinking about leaving your current job in the next year? (NQ5.1)

- ◻ Yes (1)
- ◻ Maybe (2)
- ◻ No (3)

If you answered YES or MAYBE, please go to Question 56.1.
If you answered NO, please go to Question 57

56.a Since you are thinking about leaving your current job in the next year, what would be your next career move? (NQ5.a)

- ◻ Take another job at my current employer but different practice setting (1)
- ◻ Take another job at the same kind of facility. (2)
- ◻ Take another job in a different care delivery context. (3)
- ◻ Take another job outside of nursing or midwifery. (4)
- ◻ Other: ____(5)
- ◻ Prefer not to say. (6)

56.b. The career move may involve moving: **(NQ5.b)**

- □ From an urban area to a suburban area (1)
- □ From an urban area to a rural area (2)
- □ From a suburban area to a rural area (3)
- □ From a suburban area to an urban area (4)
- □ From a rural area to a suburban area (5)
- □ From a rural area to an urban area (6)
- □ Not sure (7)
- □ Prefer not to say (8)

56.c. Would your career move involve moving to another country? **(NQ5.c)**

- □ Yes (1)
- □ No (2)
- □ Not sure (3)

57. Are you thinking about leaving the nursing or midwifery profession in the next year? **(NQ5.2)**

- □ Yes (1)
- □ No (2)
- □ Not sure (3)

< GO ON TO THE NEXT PAGE>

SECTION ON PATIENT CARE MANAGEMENT AND TREATMENT OBSERVATIONS

The following questions will ask you about your experiences with the treatment and management of people infected with the COVID-19.

58. Please list any education or training you received on the job to help you manage people infected with COVID-19 during the pandemic. **(Q30)**

59. What nursing/midwifery interventions and other treatments have you observed that improve outcomes for people infected with COVID-19? **(Q57)**

60. What nursing/midwifery interventions and other treatments have you observed that <u>DO NOT improve outcomes</u> for hospitalized people infected with COVID-19? **(Q58)**

61. Did you receive adequate training and education about the use of personal protective equipment? **(Q33)**

- □ Yes (1)
- □ No (2)
- □ Not sure (3)
- □ Prefer not to say (4)

62. Did you receive any training about the spiritual, emotional, or psychological support needs of hospitalized people infected with COVID-19? **(Q36)**

- □ Yes (1)
- □ No (2)
- □ Not sure (3)
- □ Prefer not to say (4)

Please answer the following questions about the availability of resources to manage care for people infected with COVID-19. **(NQ14)**

63.a. Evidence-based medications to treat symptoms.

- □ Not available (1)
- □ Rarely available (2)
- □ Sometimes available (3)
- □ Usually available (4)
- □ Always available (5)

63.b. Personal protective equipment.

- ◻ Not available (1)
- ◻ Rarely available (2)
- ◻ Sometimes available (3)
- ◻ Usually available (4)
- ◻ Always available (5)

63.c. Intravenous infusion equipment.

- ◻ Not available (1)
- ◻ Rarely available (2)
- ◻ Sometimes available (3)
- ◻ Usually available (4)
- ◻ Always available (5)

63.d. Oxygen and related infusions equipment.

- ◻ Not available (1)
- ◻ Rarely available (2)
- ◻ Sometimes available (3)
- ◻ Usually available (4)
- ◻ Always available (5)

63.e. Mechanical ventilator.

- ◻ Not available (1)
- ◻ Rarely available (2)
- ◻ Sometimes available (3)
- ◻ Usually available (4)
- ◻ Always available (5)

64. In the last month, how often have you had a <u>patient</u> infected with COVID-19 ask you to administer treatments that are not evidence-based? (NQ16a)

- ◻ Never (1)
- ◻ Daily (2)
- ◻ Weekly (3)

65. In the last month, how often have you had a <u>family member</u> of a patient infected with COVID-19 ask you to administer treatments that are not evidence-based? (NQ16b)

- ◻ Never (1)
- ◻ Daily (2)
- ◻ Weekly (3)

66. In the last month, how often have you had a <u>physician</u> who has ordered you to administer treatments to people infected with COVID-19 that are not evidence-based? (NQ17)

- ◻ Never (1)
- ◻ Daily (2)
- ◻ Weekly (3)

SECTION ON OCCUPATIONAL HEALTH RISKS

67. Have you been confirmed to have had a COVID-19 infection at any point between March 2020 and the present? (Q24)

- ◻ Yes (1)
- ◻ No (3)

If you answered YES to Question 67, please answer questions 67.a and 67.b.

If you answered NO to Question 67, please skip to question 68.

67.a. If you responded "yes" to having had a coronavirus infection, approximately when did you last have it? (Q25) Please indicate the month(s) and year(s)

____

67.b. Did you have to work when you knew you were infected with the COVID-19 virus?

- ◻ Yes (1)
- ◻ Yes, but only because my employer said I would lose my job (2)
- ◻ No (3)

VACCINE SERIES QUESTIONS

68. Did you receive a COVID-19 vaccination? (NQ4.1)

- ◻ Yes (1)
- ◻ No (3)

If you answered YES to question 68, please go to question 69.

If you answered NO to question 68, please go to question 68.a.

68.a If you did not get a COVID-19 vaccination, please share why you did not. **(NQ4.4)**

68.b. Since you did not get a COVID-19 vaccine, how often does your employer require you to get tested? **(NQ4.5)**

- ◻ Weekly (1)
- ◻ Biweekly (2)
- ◻ Monthly (3)
- ◻ Not at all (4)
- ◻ Not currently employed (5)

69. Where you live, what percentage of people do you think are vaccinated? **(NQ4.6)**

- ◻ 0-25% (1)
- ◻ 26-50% (2)
- ◻ 51-75% (3)
- ◻ 76-100% (4)

70. Please rate your country's overall COVID-19 vaccination efforts. **(NQ4.7)**

- ◻ Excellent (1)
- ◻ Very Good (2)
- ◻ Good (3)
- ◻ Needs improvement (4)
- ◻ Poor (5)

71. Were you assigned to work in a different place in your organization (a.k.a redeployment) at any point between March 2020 and the present as part of your organization's pandemic response? **(Q35)**

- □ Yes, temporarily (1)
- □ Yes, permanently (2)
- □ No (3)
- □ Prefer not to say (4)

If you answered YES to Question 71, please go to question 71.a.

If you answered NO or "Prefer not to say" to question 71, please go to Question 72.

71.a. If you were redeployed to a different work setting in your organization, how long did you have to work there? **(NQ18)**

- □ 1 week (1)
- □ 2 weeks (2)
- □ 3 weeks (3)
- □ 4 weeks (4)
- □ 5 weeks (5)
- □ 6 weeks (6)
- □ 7 weeks (7)
- □ 8 weeks (8)
- □ 9 weeks or more (9)
- □ Still redeployed (10)
- □ Prefer not to say (11)

72. Did you receive any extra pay or benefits because you were caring for people infected with COVID-19? **(Q38)**

- □ Yes. Please describe them below. (1)
  ____
- □ No (2)
- □ Not sure (3)
- □ Prefer not to say (4)

73. Have you had any benefit reductions due to the pandemic, e.g. reductions in retirement/pension contributions, reduced wages, frozen wages, changes in health insurance, etc.? **(Q39)**

- ◻ Yes. Please describe in the space below. (1)

  ____

  ____

  ____

- ◻ No (2)

74. Is COVID-19 considered as a workplace acquired infection by your employer? **(Q41)**

- ◻ Yes (1)
- ◻ No (2)
- ◻ Not sure (3)
- ◻ Prefer not to say (4)

75. Have you been given the option to obtain alternate housing so you can protect your family from getting an infection? **(Q42)**

- ◻ Yes (1)
- ◻ No (2)
- ◻ Not sure (3)
- ◻ Prefer not to say (4)

76. Right now, are you living in another place that is not your home to protect the people you live with from getting infected with COVID-19? **(Q47)**

- ◻ Yes (1)
- ◻ No (2)
- ◻ Prefer not to say (3)

77. Do you have to take more time to <u>prepare for work</u> when caring for people infected with COVID-19? **(Q44)**

- □ Yes (1)
- □ No (2)
- □ Not sure (3)
- □ Prefer not to say (4)

If you answered YES or NOT SURE to Question 77, please go to Question 77.a.

If you answered NO or "Prefer not to say" to Question 77, please go to Question 78.

77.a. Q45 If you have to take more time to <u>prepare for work</u> when caring for people infected with COVID-19, how much longer on average? **(Q45)**

- □ Less than an hour (1)
- □ More than one hour but less than two hours (2)
- □ More than two hours (3)

78. Do you have to take more time to <u>finish your work</u> when caring for people infected with COVID-19? **(Q105)**

- □ Yes (1)
- □ No (2)
- □ Not sure (3)
- □ Prefer not to say (4)

If you answered YES or NOT SURE to Question 78, please go to Question 78.a.

If you answered NO or "Prefer not to say" to Question 78, please go to Question 79.

78.b. If you have to take more time to finish work when caring for people infected with COVID-19, including working overtime, how much longer does it take? **(Q106)**

- □ Less than an hour (1)
- □ More than one hour but less than two hours (2)
- □ More than two hours (3)

79. What is one thing you wish had been done differently that would have made the response to the pandemic better and easier for you as a nurse or midwife? (NQ8)

80. Is there anything else you would like to add about living and working during the COVID-19 pandemic? Take as much space as you need. (Q60)

<END OF SURVEY>
