# Supplementary material for: Assessing Pandemic Response Implementation and the Workforce Impact Among Nurses and Midwives Internationally: Protocol for an Exploratory, Pragmatic, Online Descriptive Study
Source: JMIR Res Protoc. 2026 Oct 27;15:e94179. doi: 10.2196/94179 (PMC13712254; doi:10.2196/94179)
Supplement: Supplementary file 2 [file resprot-v15-e94179-s002.docx]

Global Nursing & Midwifery Perspectives of Working During the COVID-19 Pandemic

Round 2 Codebook

English Survey

Table of Contents

[How to use the Codebook	11](#_Toc197344982)

[Quantitative Items	11](#_Toc197344983)

[Demographics/Living and Working	11](#_Toc197344984)

[UserLanguage	11](#_Toc197344985)

[Q3	11](#_Toc197344986)

[Q5	11](#_Toc197344987)

[Q213 – United States Respondents Only	11](#_Toc197344988)

[Q215 – United States Respondents Only	11](#_Toc197344989)

[Q6	12](#_Toc197344990)

[Q214 – Guyana Respondents Only	12](#_Toc197344991)

[Q11	12](#_Toc197344992)

[Q7	12](#_Toc197344993)

[Q9	13](#_Toc197344994)

[Q9_9_TEXT	13](#_Toc197344995)

[Q10	13](#_Toc197344996)

[Well-Being During the Pandemic	13](#_Toc197344997)

[Q37	13](#_Toc197344998)

[Q43	14](#_Toc197344999)

[Q48_1	14](#_Toc197345000)

[Q48_2	14](#_Toc197345001)

[Q48_3	14](#_Toc197345002)

[Q48_4	15](#_Toc197345003)

[Q48_5	15](#_Toc197345004)

[Q48_6	15](#_Toc197345005)

[Q48_7	16](#_Toc197345006)

[Q48_8	16](#_Toc197345007)

[NQ10_1	16](#_Toc197345008)

[NQ10_2	16](#_Toc197345009)

[NQ10_3	17](#_Toc197345010)

[NQ10_4	17](#_Toc197345011)

[NQ10_5	17](#_Toc197345012)

[NQ10_6	18](#_Toc197345013)

[NQ10_7	18](#_Toc197345014)

[NQ10_8	18](#_Toc197345015)

[Q49	18](#_Toc197345016)

[Q50	19](#_Toc197345017)

[Q51	19](#_Toc197345018)

[Q52	19](#_Toc197345019)

[NQ12.a_1	20](#_Toc197345020)

[NQ12.a_2	20](#_Toc197345021)

[NQ12.a_3	20](#_Toc197345022)

[NQ12.a_4	20](#_Toc197345023)

[NQ12.a_5	21](#_Toc197345024)

[NQ12.a_6	21](#_Toc197345025)

[NQ12.b_1	21](#_Toc197345026)

[NQ12.b_2	22](#_Toc197345027)

[NQ12.b_3	22](#_Toc197345028)

[NQ12.b_4	22](#_Toc197345029)

[NQ12.b_5	23](#_Toc197345030)

[NQ12.b_6	23](#_Toc197345031)

[NQ15	23](#_Toc197345032)

[NQ15_1_TEXT	24](#_Toc197345033)

[NQ16	24](#_Toc197345034)

[NQ16_1_TEXT	24](#_Toc197345035)

[NQ17	24](#_Toc197345036)

[NQ17_1_TEXT	24](#_Toc197345037)

[Work During the Pandemic	24](#_Toc197345038)

[Q12	24](#_Toc197345039)

[NQ13	25](#_Toc197345040)

[Q13	25](#_Toc197345041)

[NQ12	25](#_Toc197345042)

[Q14	25](#_Toc197345043)

[Q14_4_TEXT	26](#_Toc197345044)

[Q14_14_TEXT	26](#_Toc197345045)

[Q14_Estonia – Estonia Respondents Only	26](#_Toc197345046)

[Q15	27](#_Toc197345047)

[Q16	27](#_Toc197345048)

[Q17	27](#_Toc197345049)

[Q17_6_TEXT	27](#_Toc197345050)

[Q18	27](#_Toc197345051)

[Q19	28](#_Toc197345052)

[NQ19	28](#_Toc197345053)

[NQ20	28](#_Toc197345054)

[NQ6	28](#_Toc197345055)

[NQ6_5_TEXT	29](#_Toc197345056)

[NQ7	29](#_Toc197345057)

[NQ7_3_TEXT	29](#_Toc197345058)

[NQ8	29](#_Toc197345059)

[NQ9	29](#_Toc197345060)

[Q40	30](#_Toc197345061)

[NQ14_1	30](#_Toc197345062)

[NQ14_2	30](#_Toc197345063)

[NQ14_3	30](#_Toc197345064)

[NQ14_4	31](#_Toc197345065)

[Burnout Assessment	31](#_Toc197345066)

[NQ3.1	31](#_Toc197345067)

[NQ3.2	31](#_Toc197345068)

[NQ3.3	31](#_Toc197345069)

[NQ3.4	32](#_Toc197345070)

[NQ3.5	32](#_Toc197345071)

[NQ3.6	32](#_Toc197345072)

[NQ3.7	33](#_Toc197345073)

[NQ3.8	33](#_Toc197345074)

[NQ3.9	33](#_Toc197345075)

[NQ3.10	33](#_Toc197345076)

[Wages and Earnings	34](#_Toc197345077)

[NQ22.1	34](#_Toc197345078)

[Q216 – Thailand Respondents Only	34](#_Toc197345079)

[NQ22.2	34](#_Toc197345080)

[NQ22.3	34](#_Toc197345081)

[NQ22.4	34](#_Toc197345082)

[Migration/Attrition Assessment	35](#_Toc197345083)

[NQ5.1	35](#_Toc197345084)

[NQ5.a	35](#_Toc197345085)

[NQ5.b	35](#_Toc197345086)

[NQ5.c	35](#_Toc197345087)

[NQ5.2	36](#_Toc197345088)

[Treatment/Management of the COVID-19 Patient	36](#_Toc197345089)

[Q33	36](#_Toc197345090)

[Q36	36](#_Toc197345091)

[NQ23_1	36](#_Toc197345092)

[NQ23_2	37](#_Toc197345093)

[NQ23_3	37](#_Toc197345094)

[NQ23_4	37](#_Toc197345095)

[NQ23_5	38](#_Toc197345096)

[NQ24	38](#_Toc197345097)

[Q101	38](#_Toc197345098)

[NQ25	38](#_Toc197345099)

[Q24	39](#_Toc197345100)

[Q25	39](#_Toc197345101)

[Q28	39](#_Toc197345102)

[Vaccine Series Questions	39](#_Toc197345103)

[NQ4.1	39](#_Toc197345104)

[NQ4.4	39](#_Toc197345105)

[NQ4.5	40](#_Toc197345106)

[NQ4.6	40](#_Toc197345107)

[NQ4.7	40](#_Toc197345108)

[Pandemic Response and Work Environment	40](#_Toc197345109)

[Q35	40](#_Toc197345110)

[NQ18	41](#_Toc197345111)

[Q38	41](#_Toc197345112)

[Q38_1_TEXT	41](#_Toc197345113)

[Q39	42](#_Toc197345114)

[Q39_1_TEXT	42](#_Toc197345115)

[Q41	42](#_Toc197345116)

[Q42	42](#_Toc197345117)

[Q47	42](#_Toc197345118)

[Q44	43](#_Toc197345119)

[Q45	43](#_Toc197345120)

[Q105	43](#_Toc197345121)

[Q106	43](#_Toc197345122)

[Qualitative Items	44](#_Toc197345123)

[Sources of Stress	44](#_Toc197345124)

[Q59	44](#_Toc197345125)

[NQ1	44](#_Toc197345126)

[Experiences of Violence and Aggression	44](#_Toc197345127)

[Q43_1_TEXT	44](#_Toc197345128)

[Pandemic-Related Practice Changes	44](#_Toc197345129)

[Q54	44](#_Toc197345130)

[Q55	45](#_Toc197345131)

[Q56	45](#_Toc197345132)

[Nursing Care of the COVID-19 Patient	45](#_Toc197345133)

[Q30	45](#_Toc197345134)

[Q57	45](#_Toc197345135)

[Q58	45](#_Toc197345136)

[Desired Changes and Additional Thoughts	45](#_Toc197345137)

[NQ8.1	45](#_Toc197345138)

[Q60	46](#_Toc197345139)

[Survey Items	46](#_Toc197345140)

[StartDate	46](#_Toc197345141)

[EndDate	46](#_Toc197345142)

[Status	46](#_Toc197345143)

[Progress	46](#_Toc197345144)

[Duration (in seconds)	46](#_Toc197345145)

[Finished	47](#_Toc197345146)

[RecordedDate	47](#_Toc197345147)

[Response ID	47](#_Toc197345148)

[DistributionChannel	47](#_Toc197345149)

[UserLanguage	47](#_Toc197345150)

[Q2	47](#_Toc197345151)

[Q3	48](#_Toc197345152)

[Q5	48](#_Toc197345153)

[Q213 – United States Respondents Only	48](#_Toc197345154)

[Q215 – United States Respondents Only	48](#_Toc197345155)

[Q6	48](#_Toc197345156)

[Q214 – Guyana Respondents Only	48](#_Toc197345157)

[Q11	49](#_Toc197345158)

[Q7	49](#_Toc197345159)

[Q9	49](#_Toc197345160)

[Q9_9_TEXT	50](#_Toc197345161)

[Q10	50](#_Toc197345162)

[Q59	50](#_Toc197345163)

[NQ1	50](#_Toc197345164)

[Q37	50](#_Toc197345165)

[Q43	51](#_Toc197345166)

[Q43_1_TEXT	51](#_Toc197345167)

[Q48_1	51](#_Toc197345168)

[Q48_2	51](#_Toc197345169)

[Q48_3	52](#_Toc197345170)

[Q48_4	52](#_Toc197345171)

[Q48_5	52](#_Toc197345172)

[Q48_6	52](#_Toc197345173)

[Q48_7	53](#_Toc197345174)

[Q48_8	53](#_Toc197345175)

[NQ10_1	53](#_Toc197345176)

[NQ10_2	54](#_Toc197345177)

[NQ10_3	54](#_Toc197345178)

[NQ10_4	54](#_Toc197345179)

[NQ10_5	54](#_Toc197345180)

[NQ10_6	55](#_Toc197345181)

[NQ10_7	55](#_Toc197345182)

[NQ10_8	55](#_Toc197345183)

[Q49	56](#_Toc197345184)

[Q50	56](#_Toc197345185)

[Q50_7_TEXT	56](#_Toc197345186)

[Q51	56](#_Toc197345187)

[Q51_7_TEXT	57](#_Toc197345188)

[Q52	57](#_Toc197345189)

[Q52_1_TEXT	57](#_Toc197345190)

[Q52_2_TEXT	57](#_Toc197345191)

[NQ12.a_1	58](#_Toc197345192)

[NQ12.a_2	58](#_Toc197345193)

[NQ12.a_3	58](#_Toc197345194)

[NQ12.a_4	58](#_Toc197345195)

[NQ12.a_5	59](#_Toc197345196)

[NQ12.a_6	59](#_Toc197345197)

[NQ12.b_1	59](#_Toc197345198)

[NQ12.b_2	60](#_Toc197345199)

[NQ12.b_3	60](#_Toc197345200)

[NQ12.b_4	60](#_Toc197345201)

[NQ12.b_5	61](#_Toc197345202)

[NQ12.b_6	61](#_Toc197345203)

[NQ15	61](#_Toc197345204)

[NQ15_1_TEXT	62](#_Toc197345205)

[NQ16	62](#_Toc197345206)

[NQ16_1_TEXT	62](#_Toc197345207)

[NQ17	62](#_Toc197345208)

[NQ17_1_TEXT	62](#_Toc197345209)

[Q54	62](#_Toc197345210)

[Q55	63](#_Toc197345211)

[Q56	63](#_Toc197345212)

[Q12	63](#_Toc197345213)

[NQ13	63](#_Toc197345214)

[Q13	63](#_Toc197345215)

[NQ12	64](#_Toc197345216)

[Q14	64](#_Toc197345217)

[Q14_4_TEXT	64](#_Toc197345218)

[Q14_14_TEXT	65](#_Toc197345219)

[Q14_Estonia – Estonia Respondents Only	65](#_Toc197345220)

[Q15	65](#_Toc197345221)

[Q16	65](#_Toc197345222)

[Q17	66](#_Toc197345223)

[Q17_6_TEXT	66](#_Toc197345224)

[Q18	66](#_Toc197345225)

[Q19	66](#_Toc197345226)

[NQ19	67](#_Toc197345227)

[NQ20	67](#_Toc197345228)

[NQ6	67](#_Toc197345229)

[NQ6_5_TEXT	67](#_Toc197345230)

[NQ7	67](#_Toc197345231)

[NQ7_3_TEXT	68](#_Toc197345232)

[NQ8	68](#_Toc197345233)

[NQ9	68](#_Toc197345234)

[Q40	68](#_Toc197345235)

[NQ14_1	68](#_Toc197345236)

[NQ14_2	69](#_Toc197345237)

[NQ14_3	69](#_Toc197345238)

[NQ14_4	69](#_Toc197345239)

[NQ3.1	69](#_Toc197345240)

[NQ3.2	70](#_Toc197345241)

[NQ3.3	70](#_Toc197345242)

[NQ3.4	70](#_Toc197345243)

[NQ3.5	71](#_Toc197345244)

[NQ3.6	71](#_Toc197345245)

[NQ3.7	71](#_Toc197345246)

[NQ3.8	71](#_Toc197345247)

[NQ3.9	72](#_Toc197345248)

[NQ3.10	72](#_Toc197345249)

[NQ22.1	72](#_Toc197345250)

[Q216 – Thailand Respondents Only	72](#_Toc197345251)

[NQ22.2	73](#_Toc197345252)

[NQ22.3	73](#_Toc197345253)

[NQ22.4	73](#_Toc197345254)

[NQ5.1	73](#_Toc197345255)

[NQ5.a	73](#_Toc197345256)

[NQ5.b	74](#_Toc197345257)

[NQ5.c	74](#_Toc197345258)

[NQ5.2	74](#_Toc197345259)

[Q30	74](#_Toc197345260)

[Q57	75](#_Toc197345261)

[Q58	75](#_Toc197345262)

[Q33	75](#_Toc197345263)

[Q36	75](#_Toc197345264)

[NQ23_1	76](#_Toc197345265)

[NQ23_2	76](#_Toc197345266)

[NQ23_3	76](#_Toc197345267)

[NQ23_4	76](#_Toc197345268)

[NQ23_5	77](#_Toc197345269)

[NQ24	77](#_Toc197345270)

[Q101	77](#_Toc197345271)

[NQ25	77](#_Toc197345272)

[Q24	78](#_Toc197345273)

[Q25	78](#_Toc197345274)

[Q28	78](#_Toc197345275)

[NQ4.1	78](#_Toc197345276)

[NQ4.4	79](#_Toc197345277)

[NQ4.5	79](#_Toc197345278)

[NQ4.6	79](#_Toc197345279)

[NQ4.7	79](#_Toc197345280)

[Q35	80](#_Toc197345281)

[NQ18	80](#_Toc197345282)

[Q38	80](#_Toc197345283)

[Q38_1_TEXT	81](#_Toc197345284)

[Q39	81](#_Toc197345285)

[Q39_1_TEXT	81](#_Toc197345286)

[Q41	81](#_Toc197345287)

[Q42	81](#_Toc197345288)

[Q47	82](#_Toc197345289)

[Q44	82](#_Toc197345290)

[Q45	82](#_Toc197345291)

[Q105	82](#_Toc197345292)

[Q106	83](#_Toc197345293)

[NQ8.1	83](#_Toc197345294)

[Q60	83](#_Toc197345295)

# How to use the Codebook

Please note that in the Qualtrics data file, all blank responses are marked with NA. This indicates that the survey item was **not answered**, NOT that the participant chose “Not Applicable” as their response.

# Quantitative Items

## Demographics/Living and Working

### UserLanguage

Column Letter: J

Meaning: User Language

Data Type: Categorical

Acceptable Responses: English, Albanian, Arabic, Bahasa Indonesia, Bahasa Malaysia, Simplified Chinese, Estonian, French, Georgian, German, Greek, Haitian Creole, Hebrew, Italian, Khmer, Korean, Latvian, Lithuanian, Mongolian, Polish, Portuguese, Russian, Slovenian, Spanish, Swahili, Thai, Turkish, Ukrainian, Urdu

### Q3

Column Letter: Q

Meaning: Please indicate your profession:

Data Type: Categorical

Acceptable Responses: 1, 2

1: Nursing

2: Midwifery

### Q5

Column Letter: R

Meaning: What country do you live and work in?

Data Type: Categorical

Acceptable Responses: Any world country

### Q213 – United States Respondents Only

Column Letter: S

Meaning: What state do you work in?

Data Type: Categorical

Acceptable Responses: Any state in the United States

### Q215 – United States Respondents Only

Column Letter: T

Meaning: Do you live in the same state you work in?

Data Type: Categorical

Acceptable Responses:

1. Yes
2. No

### Q6

Column Letter: U

Meaning: In what country did you receive your nursing or midwifery education?

Data Type: Categorical

Acceptable Responses: Any world country

### Q214 – Guyana Respondents Only

Column Letter: V

Meaning: What region in Guyana are you from?

Data Type: Categorical

Acceptable Responses:

- Region 1
- Region 2
- Region 3
- Region 4
- Region 5
- Region 6
- Region 7
- Region 8
- Region 9
- Region 10

### Q11

Column Letter: W

Meaning: What is your sexual orientation?

Data Type: Categorical

Acceptable Responses: 1, 2, 3, 4, 5

1: Heterosexual

2: Homosexual

3: Bisexual

4: Other

5: Prefer not to Respond.

### Q7

Column Letter: X

Meaning: What is your age?

Cases Documented:

Data Type: Nominal

### Q9

Column Letter: Y

Meaning: What is your heritage?

Data Type: Categorical

Acceptable Responses:

1. Asian or Pacific Islander
2. African origin
3. Latin American
4. Caribbean
5. European
6. Indigenous origins
7. Middle Eastern
8. Mixed Heritage
9. Other
10. Prefer not to respond

### Q9_9_TEXT

Column Letter: Z

Meaning: What is your heritage? - Other – Text

Data Type: Qualitative

Acceptable Responses: Responses are written.

### Q10

Column Letter: AA

Meaning: What is your gender?

Data Type: Qualitative

Acceptable Responses: Responses are written.

## Well-Being During the Pandemic

### Q37

Column Letter: AD

Meaning: Did (does) your employer provide appropriate mental health support

services for employees?

Data Type: Categorical

Acceptable Responses: 1, 2, 3, 4, 5

1. Yes
2. Sort of
3. No
4. Not sure
5. Prefer not to say

### Q43

Column Letter: AE

Meaning: During the last two years, as a nurse or midwife, have you experienced any anger or aggression from the public? - Selected Choice

Cases Documented: 8010

Data Type: Categorical

Acceptable Responses: 1, 2, 3, 4

1. Yes. Please describe in the space below.
2. No
3. Not sure
4. Prefer not to say

### Q48_1

Column Letter: AG

Meaning: Have you experienced any of the following symptoms at work on a regular basis since the pandemic began: - Anxiety

Data Type: Categorical

Acceptable Responses: 1, 2, 3, 4, 5

1. Yes
2. Sometimes
3. No
4. Not sure
5. Prefer not to say

### Q48_2

Column Letter: AH

Meaning: Have you experienced any of the following symptoms at work on a regular basis since the pandemic began: - Depression

Data Type: Categorical

Acceptable Responses: 1, 2, 3, 4, 5

1. Yes
2. Sometimes
3. No
4. Not sure
5. Prefer not to say

### Q48_3

Column Letter: AI

Meaning: Have you experienced any of the following symptoms at work on a regular basis since the pandemic began: - Feeling overwhelmed

Data Type: Categorical

Acceptable Responses: 1, 2, 3, 4, 5

1. Yes
2. Sometimes
3. No
4. Not sure
5. Prefer not to say

### Q48_4

Column Letter: AJ

Meaning: Have you experienced any of the following symptoms at work on a regular basis since the pandemic began: - Feeling sad frequently

Data Type: Categorical

Acceptable Responses: 1, 2, 3, 4, 5

1. Yes
2. Sometimes
3. No
4. Not sure
5. Prefer not to say

### Q48_5

Column Letter: AK

Meaning: Have you experienced any of the following symptoms at work on a regular basis since the pandemic began: - Difficulty concentrating

Data Type: Categorical

Acceptable Responses: 1, 2, 3, 4, 5

1. Yes
2. Sometimes
3. No
4. Not sure
5. Prefer not to say

### Q48_6

Column Letter: AL

Meaning: Have you experienced any of the following symptoms at work on a regular basis since the pandemic began: - Feeling tired

Data Type: Categorical

Acceptable Responses: 1, 2, 3, 4, 5

1. Yes
2. Sometimes
3. No
4. Not sure
5. Prefer not to say

### Q48_7

Column Letter: AM

Meaning: Have you experienced any of the following symptoms at work on a regular basis since the pandemic began: - Feeling irritable

Data Type: Categorical

Acceptable Responses: 1, 2, 3, 4, 5

1. Yes
2. Sometimes
3. No
4. Not sure
5. Prefer not to say

### Q48_8

Column Letter: AN

Meaning: Have you experienced any of the following symptoms at work on a regular basis since the pandemic began: - Hard to concentrate

Data Type: Categorical

Acceptable Responses: 1, 2, 3, 4, 5

1. Yes
2. Sometimes
3. No
4. Not sure
5. Prefer not to say

### NQ10_1

Column Letter: AO

Meaning: Have you regularly experienced any of the following symptoms after work when at home: - Anxiety

Data Type: Categorical

Acceptable Responses: 1, 2, 3, 4, 5

1. Yes
2. Sometimes
3. No
4. Not sure
5. Prefer not to say

### NQ10_2

Column Letter: AP

Meaning: Have you regularly experienced any of the following symptoms after work when at home: - Depression

Data Type: Categorical

Acceptable Responses: 1, 2, 3, 4, 5

1. Yes
2. Sometimes
3. No
4. Not sure
5. Prefer not to say

### NQ10_3

Column Letter: AQ

Meaning: Have you regularly experienced any of the following symptoms after work when at home: - Feeling overwhelmed

Data Type: Categorical

Acceptable Responses: 1, 2, 3, 4, 5

1. Yes
2. Sometimes
3. No
4. Not sure
5. Prefer not to say

### NQ10_4

Column Letter: AR

Meaning: Have you regularly experienced any of the following symptoms after work when at home: - Feeling sad frequently

Data Type: Categorical

Acceptable Responses: 1, 2, 3, 4, 5

1. Yes
2. Sometimes
3. No
4. Not sure
5. Prefer not to say

### NQ10_5

Column Letter: AS

Meaning: Have you regularly experienced any of the following symptoms after work when at home: - Difficulty concentrating

Data Type: Categorical

Acceptable Responses: 1, 2, 3, 4, 5

1. Yes
2. Sometimes
3. No
4. Not sure
5. Prefer not to say

### NQ10_6

Column Letter: AT

Meaning: Have you regularly experienced any of the following symptoms after work when at home: - Feeling tired

Data Type: Categorical

Acceptable Responses: 1, 2, 3, 4, 5

1. Yes
2. Sometimes
3. No
4. Not sure
5. Prefer not to say

### NQ10_7

Column Letter: AU

Meaning: Have you regularly experienced any of the following symptoms after work when at home: - Feeling irritable

Data Type: Categorical

Acceptable Responses: 1, 2, 3, 4, 5

1. Yes
2. Sometimes
3. No
4. Not sure
5. Prefer not to say

### NQ10_8

Column Letter: AV

Meaning: Have you regularly experienced any of the following symptoms after work when at home: - Hard to concentrate

Data Type: Categorical

Acceptable Responses: 1, 2, 3, 4, 5

1. Yes
2. Sometimes
3. No
4. Not sure
5. Prefer not to say

### Q49

Column Letter: AW

Meaning: Have you sought professional help for the symptoms?

Data Type: Categorical

Acceptable Responses: 1, 2, 3

1. Yes
2. No
3. Prefer not to say

### Q50

Column Letter: AX

Meaning: Since you have sought help for your symptoms related to anxiety, depression, or being overwhelmed, from whom do you or did you receive professional help? - Selected Choice

Data Type: Categorical

Acceptable Responses: 1, 2, 3, 4, 5, 6, 7

1. Workplace counselor
2. Psychologist
3. Social work
4. Pastoral care
5. Psychiatric mental health nurse practitioner
6. Psychiatrist
7. Other

### Q51

Column Letter: AZ

Meaning: If you have not sought help for your symptoms related to anxiety, depression, or being overwhelmed, why not? Please check all that apply. - Selected Choice

Data Type: Categorical

Acceptable Responses: 1, 2, 3, 4, 5, 6, 7

1. There are no mental health services near where I live or work.
2. I don’t know where to start looking.
3. My insurance does not cover mental health counseling.
4. It is too expensive for me right now.
5. Support from my coworkers, friends, or family is enough for now.
6. I am afraid of being stigmatized.
7. Other

### Q52

Column Letter: BB

Meaning: Have your personal relationships changed, such as with family members or partners, because of your work with people infected with COVID-19? - Selected Choice

Data Type: Categorical

Acceptable Responses: 1, 2, 3, 4

1. Yes. How? Please describe.
2. Somewhat. How? Please describe.
3. No.
4. Prefer not to say.

### NQ12.a_1

Column Letter: BE

Meaning: Many people report different self-care strategies for getting through the pandemic. Compared to what you did before the pandemic, what are you doing now to care for yourself?-Exercise

Data Type: Categorical

Acceptable Responses: 1, 2, 3, 4, 5

1. Doing more or better
2. About the same
3. Doing less or worse
4. Prefer not to say
5. Do not use

### NQ12.a_2

Column Letter: BF

Meaning: Many people report different self-care strategies for getting through the pandemic. Compared to what you did before the pandemic, what are you doing now to care for yourself?-Eating habits

Data Type: Categorical

Acceptable Responses: 1, 2, 3, 4, 5

1. Doing more or better
2. About the same
3. Doing less or worse
4. Prefer not to say
5. Do not use

### NQ12.a_3

Column Letter: BG

Meaning: Many people report different self-care strategies for getting through the pandemic. Compared to what you did before the pandemic, what are you doing now to care for yourself?-Spiritual practices

Data Type: Categorical

Acceptable Responses: 1, 2, 3, 4, 5

1. Doing more or better
2. About the same
3. Doing less or worse
4. Prefer not to say
5. Do not use

### NQ12.a_4

Column Letter: BH

Meaning: Many people report different self-care strategies for getting through the pandemic. Compared to what you did before the pandemic, what are you doing now to care for yourself?-Building new, supportive relationships

Data Type: Categorical

Acceptable Responses: 1, 2, 3, 4, 5

1. Doing more or better
2. About the same
3. Doing less or worse
4. Prefer not to say
5. Do not use

### NQ12.a_5

Column Letter: BI

Meaning: Many people report different self-care strategies for getting through the pandemic. Compared to what you did before the pandemic, what are you doing now to care for yourself?-Talking with family

Data Type: Categorical

Acceptable Responses: 1, 2, 3, 4, 5

1. Doing more or better
2. About the same
3. Doing less or worse
4. Prefer not to say
5. Do not use

### NQ12.a_6

Column Letter: BJ

Meaning: Many people report different self-care strategies for getting through the pandemic. Compared to what you did before the pandemic, what are you doing now to care for yourself?-Support from online communities

Data Type: Categorical

Acceptable Responses: 1, 2, 3, 4, 5

1. Doing more or better
2. About the same
3. Doing less or worse
4. Prefer not to say
5. Do not use

### NQ12.b_1

Column Letter: BK

Meaning: Many people report other coping strategies for getting through the pandemic. Compared to what you did before the pandemic, what are you doing now to cope? - Smoking tobacco

Data Type: Categorical

Acceptable Responses: 1, 2, 3, 4, 5, 6

1. More
2. About the same
3. Less
4. Quit
5. Not applicable/Do not use
6. Prefer not to say

### NQ12.b_2

Column Letter: BL

Meaning: Many people report other coping strategies for getting through the pandemic. Compared to what you did before the pandemic, what are you doing now to cope? - Drinking alcohol

Data Type: Categorical

Acceptable Responses: 1, 2, 3, 4, 5, 6

1. More
2. About the same
3. Less
4. Quit
5. Not applicable/Do not use
6. Prefer not to say

### NQ12.b_3

Column Letter: BM

Meaning: Many people report other coping strategies for getting through the pandemic. Compared to what you did before the pandemic, what are you doing now to cope? - Smoking marijuana

Data Type: Categorical

Acceptable Responses: 1, 2, 3, 4, 5, 6

1. More
2. About the same
3. Less
4. Quit
5. Not applicable/Do not use
6. Prefer not to say

### NQ12.b_4

Column Letter: BN

Meaning: Many people report other coping strategies for getting through the pandemic. Compared to what you did before the pandemic, what are you doing now to cope? - Injection drug use

Data Type: Categorical

Acceptable Responses: 1, 2, 3, 4, 5, 6

1. More
2. About the same
3. Less
4. Quit
5. Not applicable/Do not use
6. Prefer not to say

### NQ12.b_5

Column Letter: BO

Meaning: Many people report other coping strategies for getting through the pandemic. Compared to what you did before the pandemic, what are you doing now to cope? - Narcotics use

Data Type: Categorical

Acceptable Responses: 1, 2, 3, 4, 5, 6

1. More
2. About the same
3. Less
4. Quit
5. Not applicable/Do not use
6. Prefer not to say

### NQ12.b_6

Column Letter: BP

Meaning: Many people report other coping strategies for getting through the pandemic. Compared to what you did before the pandemic, what are you doing now to cope? - Cocaine use

Data Type: Categorical

Acceptable Responses: 1, 2, 3, 4, 5, 6

1. More
2. About the same
3. Less
4. Quit
5. Not applicable/Do not use
6. Prefer not to say

### NQ15

Column Letter: BQ

Meaning: Did any family members die of COVID-19? If yes, how many?

Data Type: Categorical

Acceptable Responses: 1, 2

1. Yes
2. No

### NQ15_1_TEXT

Column Letter: BR

Meaning: Did any family members die of COVID-19? If yes, how many? - Yes - Text

Data Type: Qualitative

Acceptable Responses: Responses are written.

### NQ16

Column Letter: BS

Meaning: Did you have any friends die of COVID-19? If yes, how many? - Selected Choice

Data Type: Categorical

Acceptable Responses: 1, 2

1. Yes
2. No

### NQ16_1_TEXT

Column Letter: BR

Meaning: Did you have any friends die of COVID-19? If yes, how many? - Yes - Text

Data Type: Qualitative

Acceptable Responses: Responses are written.

### NQ17

Column Letter: BU

Meaning: Did you have any co-workers die of COVID-19? If yes, how many? - Selected Choice

Data Type: Categorical

Acceptable Responses: 1, 2

1. Yes
2. No

### NQ17_1_TEXT

Column Letter: BV

Meaning: Did you have any co-workers die of COVID-19? If yes, how many? - Yes - Text

Data Type: Qualitative

Acceptable Responses: Responses are written.

## Work During the Pandemic

### Q12

Column Letter: BZ

Meaning: How many years have you worked as a nurse or midwife?

Data Type: Categorical

Acceptable Responses: 1, 2, 3, 4, 5

1. 0-3 years
2. 4-6 years
3. 7-10 years
4. 11+ years
5. Prefer not to say

### NQ13

Column Letter: CA

Meaning: At what age do you plan to retire?

Data Type: Qualitative

Acceptable Responses: Responses are written.

### Q13

Column Letter: CB

Meaning: What population is the focus of your work? You may select more than one answer.

Data Type: Categorical

Acceptable Responses: 1, 2, 3, 4

1. Mothers and/or children under 5 years of age.
2. Children ages 6 to 18 years of age.
3. Adults between 19 and 64 years of age.
4. Older adults aged 65 years or more.

### NQ12

Column Letter: CC

Meaning: Which option best describes your role in nursing or midwifery?

Data Type: Categorical

Acceptable Responses: 1, 2, 3, 4

1. Practitioner or frontline worker
2. Educator
3. Management/Administrator
4. Other

### Q14

Column Letter: CD

Meaning: What is your area of clinical practice? Choose the closest match.- Selected Choice

Data Type: Categorical

Acceptable Responses: 1-10, 13-16

1. Intensive care unit/ward
2. Intermediate or step-down unit/ward
3. Emergency department
4. Medical surgical: please specify below.
5. General medicine
6. General Surgery
7. Labor & Delivery/Obstetrics/Maternal-Child Health
8. Psychiatric & mental health nursing
9. Operating Room/Theatre
10. Long term care, physical rehabilitation, or nursing home
13. Public Health/Community Health/Health Promotion
14. Other
15. Prefer not to say
16. Oncology

### Q14_4_TEXT

Column Letter: CE

Meaning: What is your area of clinical practice? Choose the closest match. - Medical-surgical combination: Please specify below. - Text

Data Type: Qualitative

Acceptable Responses: Responses are written.

### Q14_14_TEXT

Column Letter: CF

Meaning: What is your area of clinical practice? Choose the closest match. - Other - Text

Data Type: Qualitative

Acceptable Responses: Responses are written.

### Q14_Estonia – Estonia Respondents Only

Column Letter: CG

Meaning: What is your area of clinical practice? Choose the closest match.

Data Type: Categorical

Acceptable Responses: 1, 4-15

1. Primary health care
4. Labor & delivery/obstetrics/maternal-child health
5. Long-term care and nursing care in a care home
6. Outpatient clinic
7. Ambulance
8. Emergency medicine department
9. Intensive care
10. Operating room
11. Surgical care
12. Internal medicine department
13. Rehab
14. Other
15. Prefer not to say

### Q15

Column Letter: CH

Meaning: What nursing or midwifery degree do you hold?

Data Type: Categorical

Acceptable Responses: 1-5

1. Technical, enrolled, diploma, or associate degree
2. Bachelor’s
3. Master’s
4. Doctorate
5. Prefer not to respond

### Q16

Column Letter: CI

Meaning: Do you have a specialty certification?

Data Type: Categorical

Acceptable Responses: 1, 2

1. Yes
2. No

### Q17

Column Letter: CJ

Meaning: What type of organization do you work for? - Selected choice

Data Type: Categorical

Acceptable Responses: 1, 2, 3, 4, 5, 6

1. Primary Care
2. Ambulatory Care
3. Hospital
4. Home health care
5. Other
6. Prefer not to say

### Q17_6_TEXT

Column Letter: CK

Meaning: What type of organization do you work for? - Other - Text

Data Type: Qualitative

Acceptable Responses: Responses are written.

### Q18

Column Letter: CL

Meaning: Is the institution affiliated with a university or school of nursing?

Data Type: Categorical

Acceptable Responses: 1, 2, 3, 4

1. Yes
2. No
3. Not sure.
4. Prefer not to say

### Q19

Column Letter: CM

Meaning: Where is your place of work?

Data Type: Categorical

Acceptable Responses: 1, 2, 3, 4

1. Urban setting
2. Suburban setting
3. Rural setting
4. Prefer not to say

### NQ19

Column Letter: CN

Meaning: What type of organization is your employer?

Data Type: Categorical

Acceptable Responses: 1, 2, 3, 4, 5, 6

1. Public
2. Not for Profit
3. Non-governmental organization
4. Private
5. Other
6. Prefer not to say

### NQ20

Column Letter: CO

Meaning: Are you a member of a labor union?

Data Type: Categorical

Acceptable Responses: 1, 2

1. Yes
2. No

### NQ6

Column Letter: CP

Meaning: What is your employment status?

Data Type: Categorical

Acceptable Responses: 1, 2, 3, 4, 5, 6

1. Full-time
2. Part-time (<24h/week)
3. Temporary contract
4. Per-diem
5. Other:

### NQ6_5_TEXT

Column Letter: CQ

Meaning: What is your employment status? - Other: - Text

Data Type: Qualitative

Acceptable Responses: Responses are written.

### NQ7

Column Letter: CR

Meaning: Since you are on a temporary or per-diem contract, is that:

Data Type: Categorical

Acceptable Responses: 1, 2, 3

1. By choice
2. Your only option
3. Other

### NQ7_3_TEXT

Column Letter: CS

Meaning: Since you are on a temporary or per-diem contract, is that: - Other: - Text

Data Type: Qualitative

Acceptable Responses: Responses are written.

### NQ8

Column Letter: CT

Meaning: Do you have more than one job in nursing or midwifery right now?

Data Type: Categorical

Acceptable Responses: 1, 2

1. Yes
2. No

### NQ9

Column Letter: CU

Meaning: Do you have another job outside of nursing or midwifery right now?

Data Type: Categorical

Acceptable Responses: 1, 2

1. Yes
2. No

### Q40

Column Letter: CV

Meaning: At any point between March 2020 and the present, were you furloughed or laid off from a nursing or midwifery job?

Data Type: Categorical

Acceptable Responses: 1, 2

1. Yes
2. No

### NQ14_1

Column Letter: CW

Meaning: When you were furloughed or laid off from your nursing or midwifery job, how long did it take you to: – Return to your original job

Data Type: Categorical

Acceptable Responses: 1, 2, 3, 4

1. 1 week to 3 months
2. 4-6 months
3. 7+ months
4. Not applicable

### NQ14_2

Column Letter: CX

Meaning: When you were furloughed or laid off from your nursing or midwifery job, how long did it take you to: – Find temporary employment as a nurse or midwife

Data Type: Categorical

Acceptable Responses: 1, 2, 3, 4

1. 1 week to 3 months
2. 4-6 months
3. 7+ months
4. Not Applicable

### NQ14_3

Column Letter: CY

Meaning: When you were furloughed or laid off from your nursing or midwifery job, how long did it take you to: – Find temporary employment outside of nursing or midwifery

Data Type: Categorical

Acceptable Responses: 1, 2, 3, 4

1. 1 week to 3 months
2. 4-6 months
3. 7+ months
4. Not Applicable

### NQ14_4

Column Letter: CZ

Meaning: When you were furloughed or laid off from your nursing or midwifery job, how long did it take you to: – Find part-time or full-time employment in nursing or midwifery

Data Type: Categorical

Acceptable Responses: 1, 2, 3

1. 1 week to 3 months
2. 4-6 months
3. 7+ months
4. Not applicable

## Burnout Assessment

### NQ3.1

Column Letter: DA

Meaning: Overall, I am satisfied with my current job.

Data Type: Categorical

Acceptable Responses: 1, 2, 3, 4, 5

1. Strongly Disagree
2. Disagree
3. Neither agree nor disagree
4. Agree
5. Strongly agree

### NQ3.2

Column Letter: DB

Meaning: Using your own definition of “burnout”, please choose one of the answers below.

Data Type: Categorical

Acceptable Responses: 1, 2, 3, 4, 5

1. I enjoy my work. I have no symptoms of burnout.
2. I am under stress, and don’t always have as much energy as I did, but I don’t feel burned out.
3. I am definitely burning out and have one or more symptoms of burnout, e.g. emotional exhaustion.
4. The symptoms of burnout that I’m experiencing won’t go away. I think about work frustrations a lot.
5. I feel completely burned out. I am at the point where I may need to seek help.

### NQ3.3

Column Letter: DC

Meaning: My professional values are well aligned with those of my department leaders.

Data Type: Categorical

Acceptable Responses: 1, 2, 3, 4, 5

1. Strongly Disagree
2. Disagree
3. Neither agree nor disagree
4. Agree
5. Strongly agree

### NQ3.4

Column Letter: DD

Meaning: The degree to which my care team works efficiently together is:

Data Type: Categorical

Acceptable Responses: 1, 2, 3, 4, 5

1. Excellent
2. Good
3. Satisfactory
4. Low/need improvement
5. Poor

### NQ3.5

Column Letter: DE

Meaning: I feel a great deal of stress because of my job

Data Type: Categorical

Acceptable Responses: 1, 2, 3, 4, 5

1. Strongly Disagree
2. Disagree
3. Neither agree nor disagree
4. Agree
5. Strongly agree

### NQ3.6

Column Letter: DF

Meaning: I stay after my shift is done to finish documentation (paper or electronic):

Data Type: Categorical

Acceptable Responses: 1, 2, 3, 4, 5, 6

1. Every time I work
2. 2-3 times/week
3. Once per week
4. Once every two weeks
5. Once a month
6. Very rarely

### NQ3.7

Column Letter: DG

Meaning: If I have to stay after my shift ends to finish documentation, I get paid overtime.

Data Type: Categorical

Acceptable Responses: 1, 2, 3

1. Yes
2. Sometimes
3. No

### NQ3.8

Column Letter: DH

Meaning: Which answer best describes the atmosphere in your primary work area?

Data Type: Categorical

Acceptable Responses: 1, 2, 3, 4, 5

1. Calm
2. Mostly quiet with periods of busyness
3. Busy, but reasonable
4. Constant activity, occasionally very intense
5. Hectic, chaotic

### NQ3.9

Column Letter: DI

Meaning: My control over my workload is:

Data Type: Categorical

Acceptable Responses: 1, 2, 3, 4, 5

1. Excellent
2. Good
3. Satisfactory
4. Low/need improvement
5. Poor

### NQ3.10

Column Letter: DJ

Meaning: Overall, my work environment is:

Data Type: Categorical

Acceptable Responses: 1, 2, 3, 4, 5

1. Excellent
2. Very Good
3. Good
4. Not too good
5. Bad

## Wages and Earnings

### NQ22.1

Column Letter: DK

Meaning: From your primary job, what is your monthly salary? Please respond in your country's currency?

Data Type: Qualitative

Acceptable Responses: Responses are written.

### Q216 – Thailand Respondents Only

Column Letter: DL

Meaning: From your primary job, what is your monthly salary?

Data Type: Categorical

Acceptable Responses: 1, 2, 3, 4, 5

1. 12,000-20,000 baht
2. 20,001-30,000 baht
3. 30,001-40,000 baht
4. 40,001-50,000 baht
5. >50,000 baht

### NQ22.2

Column Letter: DM

Meaning: Is your monthly salary enough to keep you out of poverty?

Data Type: Categorical

Acceptable Responses: 1, 2

1. Yes
2. No

### NQ22.3

Column Letter: DN

Meaning: Is your monthly salary enough to keep your household out of poverty?

Data Type: Categorical

Acceptable Responses: 1, 2

1. Yes
2. No

### NQ22.4

Column Letter: DO

Meaning: Are you the primary wage earner in your household?

Data Type: Categorical

Acceptable Responses: 1, 2

1. Yes
2. No

## Migration/Attrition Assessment

### NQ5.1

Column Letter: DP

Meaning: Are you thinking about leaving your current job in the next year?

Data Type: Categorical

Acceptable Responses: 1, 2, 3

1. Yes
2. No
3. Maybe

### NQ5.a

Column Letter: DQ

Meaning: Since you are thinking about leaving your current job in the next year, what would be your next career move?

Data Type: Categorical

Acceptable Responses: 1, 2, 3, 4, 5, 6

1. Take another job at my current employer but different practice setting
2. Take another job at the same kind of facility.
3. Take another job in a different care delivery context
4. Take another job outside of nursing or midwifery
5. Other
6. Prefer not to say

### NQ5.b

Column Letter: DR

Meaning: The career move may involve moving:

Data Type: Categorical

Acceptable Responses: 1, 2, 3, 4, 5, 6, 7, 9

1. From an urban to a suburban area
2. From an urban area to a rural area
3. From a suburban area to an urban area
4. From a rural area to a suburban area
5. From a rural area to an urban area
6. Not sure
7. Prefer not to say
9. From a suburban area to a rural area

### NQ5.c

Column Letter: DS

Meaning: Would your career move involve moving to another country?

Data Type: Categorical

Acceptable Responses: 1, 2, 3

1. Yes
2. No
3. Maybe

### NQ5.2

Column Letter: DT

Meaning: Are you thinking about leaving the nursing or midwifery profession in the next year?

Data Type: Categorical

Acceptable Responses: 1, 2, 3

1. Yes
2. No
3. Maybe

## Treatment/Management of the COVID-19 Patient

### Q33

Column Letter: DX

Meaning: Did you receive adequate training and education about the use of personal protective equipment?

Data Type: Categorical

Acceptable Responses: 1, 2, 3, 4

1. Yes
2. No
3. Not sure
4. Prefer not to say

### Q36

Column Letter: DY

Meaning: Did you receive any training about the spiritual, emotional, or psychological support needs of hospitalized people infected with COVID-19?

Data Type: Categorical

Acceptable Responses: 1, 2, 3, 4

1. Yes
2. No
3. Not sure
4. Prefer not to say

### NQ23_1

Column: DZ

Meaning: Please answer the following questions about the availability of resources to manage care for people infected with COVID-19.- Evidence-based medications to treat symptoms

Data Type: Categorical

Acceptable Responses: 1, 2, 3, 4, 5

1. Not available
2. Rarely available
3. Sometimes available
4. Usually available
5. Always available

### NQ23_2

Column: EA

Meaning: Please answer the following questions about the availability of resources to manage care for people infected with COVID-19.- Personal protective equipment

Data Type: Categorical

Acceptable Responses: 1, 2, 3, 4, 5

1. Not available
2. Rarely available
3. Sometimes available
4. Usually available
5. Always available

### NQ23_3

Column: EB

Meaning: Please answer the following questions about the availability of resources to manage care for people infected with COVID-19.- Intravenous infusion equipment

Data Type: Categorical

Acceptable Responses: 1, 2, 3, 4, 5

1. Not available
2. Rarely available
3. Sometimes available
4. Usually available
5. Always available

### NQ23_4

Column: EC

Meaning: Please answer the following questions about the availability of resources to manage care for people infected with COVID-19.- Oxygen and related infusion equipment

Data Type: Categorical

Acceptable Responses: 1, 2, 3, 4, 5

1. Not available
2. Rarely available
3. Sometimes available
4. Usually available
5. Always available

### NQ23_5

Column: ED

Meaning: Please answer the following questions about the availability of resources to manage care for people infected with COVID-19.- Mechanical ventilator

Data Type: Categorical

Acceptable Responses: 1, 2, 3, 4, 5

1. Not available
2. Rarely available
3. Sometimes available
4. Usually available
5. Always available

### NQ24

Column Letter: EE

Meaning: In the last month, how often have you had a patient infected with COVID-19 ask you to administer treatments that are not evidence-based?

Data Type: Categorical

Acceptable Responses: 1, 2, 3

3. Never
4. Daily
5. Weekly

### Q101

Column Letter: EF

Meaning: In the last month, how often have you had family members of patients infected with COVID-19 ask you to administer treatments that are not evidence-based?

Data Type: Categorical

Acceptable Responses: 1, 2, 3

1. Never
2. Daily
3. Weekly

### NQ25

Column Letter: EG

Meaning: In the last month, how often have you had a physician who has ordered you to administer treatments to people with COVID-19 that are not evidence-based?

Data Type: Categorical

Acceptable Responses: 1, 2, 3

1. Never
2. Daily
3. Weekly

### Q24

Column Letter: EH

Meaning: Have you been confirmed to have had a COVID-19 infection at any point between March 2020 and the present?

Data Type: Categorical

Acceptable Responses: 1, 2

1. Yes
2. No

### Q25

Column Letter: EI

Meaning: If you responded "yes" to experiencing symptoms of a coronavirus infection, please describe the symptoms you experienced.

Data Type: Qualitative

Acceptable Responses: Responses are written.

### Q28

Column Letter: EJ

Meaning: Did you have to work when you knew you were infected with the COVID-19 virus?

Data Type: Categorical

Acceptable Responses: 1, 2, 3

1. Yes
2. Yes, but only because my employer said I would lose my job
3. No

## Vaccine Series Questions

### NQ4.1

Column Letter: EK

Meaning: Did you receive a COVID-19 vaccination?

Data Type: Categorical

Acceptable Responses: 1, 2

1. Yes
2. No

### NQ4.4

Column Letter: EL

Meaning: IF you did not get a COVID-19 vaccination, please share why you did not.

Data Type: Qualitative

Acceptable Responses: Responses are written.

### NQ4.5

Column Letter: EM

Meaning: Since you did not get a COVID-19 vaccine, how often does your employer require you to get tested?

Data Type: Categorical

Acceptable Responses: 1, 2, 3, 4, 5

1. Weekly
2. Biweekly
3. Monthly
4. Not at all
5. Not currently employed

### NQ4.6

Column Letter: EN

Meaning: Where you live, what percentage of people do you think are vaccinated?

Data Type: Categorical

Acceptable Responses: 1, 2

1. 0-25%
2. 26-50%
3. 51-75%
4. 76-100%

### NQ4.7

Column Letter: EO

Meaning: Please rate your country's overall COVID-19 vaccination efforts.

Data Type: Categorical

Acceptable Responses: 1, 2, 3, 4, 5

1. Excellent
2. Very good
3. Good
4. Needs improvement
5. Poor

## Pandemic Response and Work Environment

### Q35

Column Letter: EP

Meaning: Were you assigned to work in a different place in your organization (a.k.a redeployment) at any point between March 2020 and December 2023 as part of your organization’s pandemic response?

Data Type: Categorical

Acceptable Responses: 1, 2, 3, 4

1. Yes
2. No
3. Not sure
4. Prefer not to say

### NQ18

Column Letter: EQ

Meaning: If you were redeployed to a different work setting in your organization how long did you have to work there?

Data Type: Categorical

Acceptable Responses: 1, 3, 4, 5, 6, 7, 8, 9, 10, 11, 12

1. 1 week
3. 2 weeks
4. 3 weeks
5. 4 weeks
6. 5 weeks
7. 6 weeks
8. 7 weeks
9. 8 weeks
10. 9 weeks or more
11. Still redeployed
12. Prefer not to say

### Q38

Column Letter: ER

Meaning: Did you receive any extra pay or benefits because you were caring for people infected with COVID-19? - Selected Choice

Data Type: Categorical

Acceptable Responses: 1, 2, 3, 4

1. Yes. Please describe them below.
2. No
3. Not sure
4. Prefer not to say

### Q38_1_TEXT

Column Letter: ES

Meaning: Did you receive any extra pay or benefits because you were caring for people infected with COVID-19? - Yes. Please describe them below. - Text

Data Type: Qualitative

Acceptable Responses: Responses are written.

### Q39

Column Letter: ET

Meaning: Have you had any benefit reductions due to the pandemic, e.g. reductions in retirement contributions, changes in health insurance, etc.? - Selected Choice

Data Type: Categorical

Acceptable Responses: 1, 2

1. Yes. Please describe in the space below
2. No

### Q39_1_TEXT

Column Letter: EU

Meaning: Have you had any benefit reductions due to the pandemic, e.g. reductions in retirement contributions, changes in health insurance, etc.? - Selected Choice

Data Type: Qualitative

Acceptable Responses: Responses are written.

### Q41

Column Letter: EV

Meaning: Is COVID-19 considered as a workplace acquired infection by your employer?

Data Type: Categorical

Acceptable Responses: 1, 2, 3, 4

1. Yes
2. No
3. Not sure
4. Prefer not to say

### Q42

Column Letter: EW

Meaning: Have you been given the option to obtain alternate housing so you can protect your family from getting an infection?

Data Type: Categorical

Acceptable Responses: 1, 2, 3, 4

1. Yes
2. No
3. Not sure
4. Prefer not to say

### Q47

Column Letter: EX

Meaning: Right now, are you living in another place that is not your home to protect the people you live with from getting infected with COVID-19?

Data Type: Categorical

Acceptable Responses: 1, 2, 3

1. Yes
2. No
3. Prefer not to say

### Q44

Column Letter: EY

Meaning: Do you have to take more time to prepare for work when caring for people infected with COVID-19?

Data Type: Categorical

Acceptable Responses: 1, 2, 3, 4

1. Yes
2. No
3. Not sure
4. Prefer not to say

### Q45

Column Letter: EZ

Meaning: If you have to take more time to prepare for work when caring for people infected with COVID-19, how much longer on average?

Data Type: Categorical

Acceptable Responses: 1, 2, 3

1. Less than an hour
2. More than one hour but less than two hours
3. More than two hours

### Q105

Column Letter: FA

Meaning: Do you have to take more time to prepare for work when caring for people infected with COVID-19?

Data Type: Categorical

Acceptable Responses: 1, 2, 3, 4

1. Yes
2. No
3. Not sure
4. Prefer not to say

### Q106

Column Letter: FB

Meaning: If you have to take more time to prepare for work when caring for people infected with COVID-19, how much longer on average?

Data Type: Categorical

Acceptable Responses: 1, 2, 3

1. Less than an hour
2. More than one hour but less than two hours
3. More than two hours

# Qualitative Items

## Sources of Stress

### Q59

Column Letter: AB

Meaning: Please describe your main sources of work-related stress

during the pandemic. If your sources of work-related stress have changed over time, please describe how. Take as much space as you need.

Data Type: Qualitative

Acceptable Responses: Responses are written.

### NQ1

Column Letter: AC

Meaning: Please describe your main sources of personal stress during the pandemic. If your sources of personal stress have changed over time, please describe how. Take as much space as you need.

Data Type: Qualitative

Acceptable Responses: Responses are written.

## Experiences of Violence and Aggression

### Q43_1_TEXT

Column Letter: AF

Meaning: During the last two years, as a nurse or midwife, have you experienced

any anger or aggression from the public? - Yes. Please describe in the space below. - Text

Data Type: Qualitative

Acceptable Responses: Responses are written.

## Pandemic-Related Practice Changes

### Q54

Column Letter: BW

Meaning: Please describe how your practice as a nurse or midwife has changed during the pandemic.

Data Type: Qualitative

Acceptable Responses: Responses are written.

### Q55

Column Letter: BX

Meaning: Please describe how your employer has responded to the pandemic. What did they do well? What did they not do well?

Data Type: Qualitative

Acceptable Responses: Responses are written.

### Q56

Column Letter: BY

Meaning: Please tell us about how the pandemic affected teamwork in your workplace. Please share about teamwork with fellow nurses or midwives, physicians, and other allied health professionals.

Data Type: Qualitative

Acceptable Responses: Responses are written.

## Nursing Care of the COVID-19 Patient

### Q30

Column Letter: DU

Meaning: Please list any education or training you received on the job to help you manage people infected with COVID-19 during the pandemic.

Data Type: Qualitative

Acceptable Responses: Responses are written.

### Q57

Column Letter: DV

Meaning: What nursing interventions or other treatments have you observed that improve outcomes for hospitalized people infected with COVID-19?

Data Type: Qualitative

Acceptable Responses: Responses are written.

### Q58

Column Letter: DW

Meaning: What nursing interventions or other treatments have you observed that DO NOT improve outcomes for hospitalized people infected with COVID-19?

Data Type: Qualitative

Acceptable Responses: Responses are written.

## Desired Changes and Additional Thoughts

### NQ8.1

*Column Letter:* FC

*Meaning:* What is one thing you wish had been done differently that would have made the response to the pandemic better and easier for you as a nurse or midwife?

*Data Type:* Qualitative

*Acceptable Responses:* Responses are written.

### Q60

Column Letter: FD

Meaning: Is there anything else you would like to add about living and working during the COVID-19 pandemic? Take as much space as you need.

Data Type: Qualitative

Acceptable Responses: Responses are written.

# Survey Items

## StartDate

Column Letter: A

Meaning: Start Date

Acceptable Responses: Dates listed in MM/DD/YYYY hh:mm:ss

## EndDate

Column Letter: B

Meaning: End Date

Acceptable Response Range: Dates listed in MM/DD/YYYY hh:mm:ss

## Status

Column Letter: C

Meaning: Response Type

Data Type: Categorical

Acceptable Responses: IP Address, Survey Preview, Spam

## Progress

Column Letter: D

Meaning: Progress

Data Type: Ordinal

Acceptable Response Range: 0-100

## Duration (in seconds)

Column Letter: E

Meaning: Duration

## Finished

Column Letter: F

Meaning: Finished

Data Type: Categorical

Acceptable Responses:

0: False

1: True

## RecordedDate

Column Letter: G

Meaning: Recorded Date

Acceptable Response Range: Dates listed in MM/DD/YYYY hh:mm:ss

## Response ID

Column Letter: H

Meaning: Response ID

Data Type: Qualitative

Acceptable Responses: R_xxxxxxxxxxxxxxx

‘x’ represents any collection of letters and/or numbers.

## DistributionChannel

Column Letter: I

Meaning: Distribution Channel

Data Type: Categorical

Acceptable Responses: anonymous, preview, qr, socia

## UserLanguage

Column Letter: J

Meaning: User Language

Data Type: Categorical

Acceptable Responses: English, Albanian, Arabic, Bahasa Indonesia, Bahasa Malaysia, Simplified Chinese, Estonian, French, Georgian, German, Greek, Haitian Creole, Hebrew, Italian, Khmer, Korean, Latvian, Lithuanian, Mongolian, Polish, Portuguese, Russian, Slovenian, Spanish, Swahili, Thai, Turkish, Ukrainian, Urdu

## Q2

Column Letter: P

Meaning: Do you agree to participate in this study?

Data Type: Categorical

Acceptable Responses:

1: Yes

2: No

## Q3

Column Letter: Q

Meaning: Please indicate your profession:

Data Type: Categorical

Acceptable Responses: 1, 2

1: Nursing

2: Midwifery

## Q5

Column Letter: R

Meaning: What country do you live and work in?

Data Type: Categorical

Acceptable Responses: Any world country

## Q213 – United States Respondents Only

Column Letter: S

Meaning: What state do you work in?

Data Type: Categorical

Acceptable Responses: Any state in the United States

## Q215 – United States Respondents Only

Column Letter: T

Meaning: Do you live in the same state you work in?

Data Type: Categorical

Acceptable Responses:

1. Yes
2. No

## Q6

Column Letter: U

Meaning: In what country did you receive your nursing or midwifery education?

Data Type: Categorical

Acceptable Responses: Any world country

## Q214 – Guyana Respondents Only

Column Letter: V

Meaning: What region in Guyana are you from?

Data Type: Categorical

Acceptable Responses:

- Region 1
- Region 2
- Region 3
- Region 4
- Region 5
- Region 6
- Region 7
- Region 8
- Region 9
- Region 10

## Q11

Column Letter: W

Meaning: What is your sexual orientation?

Data Type: Categorical

Acceptable Responses: 1, 2, 3, 4, 5

1: Heterosexual

2: Homosexual

3: Bisexual

4: Other

5: Prefer not to Respond.

## Q7

Column Letter: X

Meaning: What is your age?

Cases Documented:

Data Type: Nominal

## Q9

Column Letter: Y

Meaning: What is your heritage?

Data Type: Categorical

Acceptable Responses:

1. Asian or Pacific Islander
2. African origin
3. Latin American
4. Caribbean
5. European
6. Indigenous origins
7. Middle Eastern
8. Mixed Heritage
9. Other
10. Prefer not to respond

## Q9_9_TEXT

Column Letter: Z

Meaning: What is your heritage? - Other – Text

Data Type: Qualitative

Acceptable Responses: Responses are written.

## Q10

Column Letter: AA

Meaning: What is your gender?

Data Type: Qualitative

Acceptable Responses: Responses are written.

## Q59

Column Letter: AB

Meaning: Please describe your main sources of work-related stress

during the pandemic. If your sources of work-related stress have changed over time, please describe how. Take as much space as you need.

Data Type: Qualitative

Acceptable Responses: Responses are written.

## NQ1

Column Letter: AC

Meaning: Please describe your main sources of personal stress during the pandemic. If your sources of personal stress have changed over time, please describe how. Take as much space as you need.

Data Type: Qualitative

Acceptable Responses: Responses are written.

## Q37

Column Letter: AD

Meaning: Did (does) your employer provide appropriate mental health support

services for employees?

Data Type: Categorical

Acceptable Responses: 1, 2, 3, 4, 5

1. Yes
2. Sort of
3. No
4. Not sure
5. Prefer not to say

## Q43

Column Letter: AE

Meaning: During the last two years, as a nurse or midwife, have you experienced any anger or aggression from the public? - Selected Choice

Cases Documented: 8010

Data Type: Categorical

Acceptable Responses: 1, 2, 3, 4

1. Yes. Please describe in the space below.
2. No
3. Not sure
4. Prefer not to say

## Q43_1_TEXT

Column Letter: AF

Meaning: During the last two years, as a nurse or midwife, have you experienced

any anger or aggression from the public? - Yes. Please describe in the space below. - Text

Data Type: Qualitative

Acceptable Responses: Responses are written.

## Q48_1

Column Letter: AG

Meaning: Have you experienced any of the following symptoms at work on a regular basis since the pandemic began: - Anxiety

Data Type: Categorical

Acceptable Responses: 1, 2, 3, 4, 5

1. Yes
2. Sometimes
3. No
4. Not sure
5. Prefer not to say

## Q48_2

Column Letter: AH

Meaning: Have you experienced any of the following symptoms at work on a regular basis since the pandemic began: - Depression

Data Type: Categorical

Acceptable Responses: 1, 2, 3, 4, 5

1. Yes
2. Sometimes
3. No
4. Not sure
5. Prefer not to say

## Q48_3

Column Letter: AI

Meaning: Have you experienced any of the following symptoms at work on a regular basis since the pandemic began: - Feeling overwhelmed

Data Type: Categorical

Acceptable Responses: 1, 2, 3, 4, 5

1. Yes
2. Sometimes
3. No
4. Not sure
5. Prefer not to say

## Q48_4

Column Letter: AJ

Meaning: Have you experienced any of the following symptoms at work on a regular basis since the pandemic began: - Feeling sad frequently

Data Type: Categorical

Acceptable Responses: 1, 2, 3, 4, 5

1. Yes
2. Sometimes
3. No
4. Not sure
5. Prefer not to say

## Q48_5

Column Letter: AK

Meaning: Have you experienced any of the following symptoms at work on a regular basis since the pandemic began: - Difficulty concentrating

Data Type: Categorical

Acceptable Responses: 1, 2, 3, 4, 5

1. Yes
2. Sometimes
3. No
4. Not sure
5. Prefer not to say

## Q48_6

Column Letter: AL

Meaning: Have you experienced any of the following symptoms at work on a regular basis since the pandemic began: - Feeling tired

Data Type: Categorical

Acceptable Responses: 1, 2, 3, 4, 5

1. Yes
2. Sometimes
3. No
4. Not sure
5. Prefer not to say

## Q48_7

Column Letter: AM

Meaning: Have you experienced any of the following symptoms at work on a regular basis since the pandemic began: - Feeling irritable

Data Type: Categorical

Acceptable Responses: 1, 2, 3, 4, 5

1. Yes
2. Sometimes
3. No
4. Not sure
5. Prefer not to say

## Q48_8

Column Letter: AN

Meaning: Have you experienced any of the following symptoms at work on a regular basis since the pandemic began: - Hard to concentrate

Data Type: Categorical

Acceptable Responses: 1, 2, 3, 4, 5

1. Yes
2. Sometimes
3. No
4. Not sure
5. Prefer not to say

## NQ10_1

Column Letter: AO

Meaning: Have you regularly experienced any of the following symptoms after work when at home: - Anxiety

Data Type: Categorical

Acceptable Responses: 1, 2, 3, 4, 5

1. Yes
2. Sometimes
3. No
4. Not sure
5. Prefer not to say

## NQ10_2

Column Letter: AP

Meaning: Have you regularly experienced any of the following symptoms after work when at home: - Depression

Data Type: Categorical

Acceptable Responses: 1, 2, 3, 4, 5

1. Yes
2. Sometimes
3. No
4. Not sure
5. Prefer not to say

## NQ10_3

Column Letter: AQ

Meaning: Have you regularly experienced any of the following symptoms after work when at home: - Feeling overwhelmed

Data Type: Categorical

Acceptable Responses: 1, 2, 3, 4, 5

1. Yes
2. Sometimes
3. No
4. Not sure
5. Prefer not to say

## NQ10_4

Column Letter: AR

Meaning: Have you regularly experienced any of the following symptoms after work when at home: - Feeling sad frequently

Data Type: Categorical

Acceptable Responses: 1, 2, 3, 4, 5

1. Yes
2. Sometimes
3. No
4. Not sure
5. Prefer not to say

## NQ10_5

Column Letter: AS

Meaning: Have you regularly experienced any of the following symptoms after work when at home: - Difficulty concentrating

Data Type: Categorical

Acceptable Responses: 1, 2, 3, 4, 5

1. Yes
2. Sometimes
3. No
4. Not sure
5. Prefer not to say

## NQ10_6

Column Letter: AT

Meaning: Have you regularly experienced any of the following symptoms after work when at home: - Feeling tired

Data Type: Categorical

Acceptable Responses: 1, 2, 3, 4, 5

1. Yes
2. Sometimes
3. No
4. Not sure
5. Prefer not to say

## NQ10_7

Column Letter: AU

Meaning: Have you regularly experienced any of the following symptoms after work when at home: - Feeling irritable

Data Type: Categorical

Acceptable Responses: 1, 2, 3, 4, 5

1. Yes
2. Sometimes
3. No
4. Not sure
5. Prefer not to say

## NQ10_8

Column Letter: AV

Meaning: Have you regularly experienced any of the following symptoms after work when at home: - Hard to concentrate

Data Type: Categorical

Acceptable Responses: 1, 2, 3, 4, 5

1. Yes
2. Sometimes
3. No
4. Not sure
5. Prefer not to say

## Q49

Column Letter: AW

Meaning: Have you sought professional help for the symptoms?

Data Type: Categorical

Acceptable Responses: 1, 2, 3

1. Yes
2. No
3. Prefer not to say

## Q50

Column Letter: AX

Meaning: Since you have sought help for your symptoms related to anxiety, depression, or being overwhelmed, from whom do you or did you receive professional help? - Selected Choice

Data Type: Categorical

Acceptable Responses: 1, 2, 3, 4, 5, 6, 7

1. Workplace counselor
2. Psychologist
3. Social work
4. Pastoral care
5. Psychiatric mental health nurse practitioner
6. Psychiatrist
7. Other

## Q50_7_TEXT

Column Letter: AY

Meaning: Since you have sought help for your symptoms related to anxiety, depression, or being overwhelmed, from whom do you or did you receive professional help? - Other – Text

Data Type: Qualitative

Acceptable Responses: Responses are written.

## Q51

Column Letter: AZ

Meaning: If you have not sought help for your symptoms related to anxiety, depression, or being overwhelmed, why not? Please check all that apply. - Selected Choice

Data Type: Categorical

Acceptable Responses: 1, 2, 3, 4, 5, 6, 7

1. There are no mental health services near where I live or work.
2. I don’t know where to start looking.
3. My insurance does not cover mental health counseling.
4. It is too expensive for me right now.
5. Support from my coworkers, friends, or family is enough for now.
6. I am afraid of being stigmatized.
7. Other

## Q51_7_TEXT

Column Letter: BA

Meaning: If you have not sought help for your symptoms related to anxiety, depression, or being overwhelmed, why not? Please check all that apply. – Other - Text

Data Type: Qualitative

Acceptable Responses: Responses are written.

## Q52

Column Letter: BB

Meaning: Have your personal relationships changed, such as with family members or partners, because of your work with people infected with COVID-19? - Selected Choice

Data Type: Categorical

Acceptable Responses: 1, 2, 3, 4

1. Yes. How? Please describe.
2. Somewhat. How? Please describe.
3. No.
4. Prefer not to say.

## Q52_1_TEXT

Column Letter: BC

Meaning: Have your personal relationships changed, such as with family members or partners, because of your work with people infected with COVID-19? - Yes. How? Please describe. – Text

Data Type: Qualitative

Acceptable Responses: Responses are written.

## Q52_2_TEXT

Column Letter: BD

Meaning: Have your personal relationships changed, such as with family members or partners, because of your work with people infected with COVID-19? - Somewhat. How? Please describe. – Text

Data Type: Qualitative

Acceptable Responses: Responses are written.

## NQ12.a_1

Column Letter: BE

Meaning: Many people report different self-care strategies for getting through the pandemic. Compared to what you did before the pandemic, what are you doing now to care for yourself?-Exercise

Data Type: Categorical

Acceptable Responses: 1, 2, 3, 4, 5

1. Doing more or better
2. About the same
3. Doing less or worse
4. Prefer not to say
5. Do not use

## NQ12.a_2

Column Letter: BF

Meaning: Many people report different self-care strategies for getting through the pandemic. Compared to what you did before the pandemic, what are you doing now to care for yourself?-Eating habits

Data Type: Categorical

Acceptable Responses: 1, 2, 3, 4, 5

1. Doing more or better
2. About the same
3. Doing less or worse
4. Prefer not to say
5. Do not use

## NQ12.a_3

Column Letter: BG

Meaning: Many people report different self-care strategies for getting through the pandemic. Compared to what you did before the pandemic, what are you doing now to care for yourself?-Spiritual practices

Data Type: Categorical

Acceptable Responses: 1, 2, 3, 4, 5

1. Doing more or better
2. About the same
3. Doing less or worse
4. Prefer not to say
5. Do not use

## NQ12.a_4

Column Letter: BH

Meaning: Many people report different self-care strategies for getting through the pandemic. Compared to what you did before the pandemic, what are you doing now to care for yourself?-Building new, supportive relationships

Data Type: Categorical

Acceptable Responses: 1, 2, 3, 4, 5

1. Doing more or better
2. About the same
3. Doing less or worse
4. Prefer not to say
5. Do not use

## NQ12.a_5

Column Letter: BI

Meaning: Many people report different self-care strategies for getting through the pandemic. Compared to what you did before the pandemic, what are you doing now to care for yourself?-Talking with family

Data Type: Categorical

Acceptable Responses: 1, 2, 3, 4, 5

1. Doing more or better
2. About the same
3. Doing less or worse
4. Prefer not to say
5. Do not use

## NQ12.a_6

Column Letter: BJ

Meaning: Many people report different self-care strategies for getting through the pandemic. Compared to what you did before the pandemic, what are you doing now to care for yourself?-Support from online communities

Data Type: Categorical

Acceptable Responses: 1, 2, 3, 4, 5

1. Doing more or better
2. About the same
3. Doing less or worse
4. Prefer not to say
5. Do not use

## NQ12.b_1

Column Letter: BK

Meaning: Many people report other coping strategies for getting through the pandemic. Compared to what you did before the pandemic, what are you doing now to cope? - Smoking tobacco

Data Type: Categorical

Acceptable Responses: 1, 2, 3, 4, 5, 6

1. More
2. About the same
3. Less
4. Quit
5. Not applicable/Do not use
6. Prefer not to say

## NQ12.b_2

Column Letter: BL

Meaning: Many people report other coping strategies for getting through the pandemic. Compared to what you did before the pandemic, what are you doing now to cope? - Drinking alcohol

Data Type: Categorical

Acceptable Responses: 1, 2, 3, 4, 5, 6

1. More
2. About the same
3. Less
4. Quit
5. Not applicable/Do not use
6. Prefer not to say

## NQ12.b_3

Column Letter: BM

Meaning: Many people report other coping strategies for getting through the pandemic. Compared to what you did before the pandemic, what are you doing now to cope? - Smoking marijuana

Data Type: Categorical

Acceptable Responses: 1, 2, 3, 4, 5, 6

1. More
2. About the same
3. Less
4. Quit
5. Not applicable/Do not use
6. Prefer not to say

## NQ12.b_4

Column Letter: BN

Meaning: Many people report other coping strategies for getting through the pandemic. Compared to what you did before the pandemic, what are you doing now to cope? - Injection drug use

Data Type: Categorical

Acceptable Responses: 1, 2, 3, 4, 5, 6

1. More
2. About the same
3. Less
4. Quit
5. Not applicable/Do not use
6. Prefer not to say

## NQ12.b_5

Column Letter: BO

Meaning: Many people report other coping strategies for getting through the pandemic. Compared to what you did before the pandemic, what are you doing now to cope? - Narcotics use

Data Type: Categorical

Acceptable Responses: 1, 2, 3, 4, 5, 6

1. More
2. About the same
3. Less
4. Quit
5. Not applicable/Do not use
6. Prefer not to say

## NQ12.b_6

Column Letter: BP

Meaning: Many people report other coping strategies for getting through the pandemic. Compared to what you did before the pandemic, what are you doing now to cope? - Cocaine use

Data Type: Categorical

Acceptable Responses: 1, 2, 3, 4, 5, 6

1. More
2. About the same
3. Less
4. Quit
5. Not applicable/Do not use
6. Prefer not to say

## NQ15

Column Letter: BQ

Meaning: Did any family members die of COVID-19? If yes, how many?

Data Type: Categorical

Acceptable Responses: 1, 2

1. Yes
2. No

## NQ15_1_TEXT

Column Letter: BR

Meaning: Did any family members die of COVID-19? If yes, how many? - Yes - Text

Data Type: Qualitative

Acceptable Responses: Responses are written.

## NQ16

Column Letter: BS

Meaning: Did you have any friends die of COVID-19? If yes, how many? - Selected Choice

Data Type: Categorical

Acceptable Responses: 1, 2

1. Yes
2. No

## NQ16_1_TEXT

Column Letter: BR

Meaning: Did you have any friends die of COVID-19? If yes, how many? - Yes - Text

Data Type: Qualitative

Acceptable Responses: Responses are written.

## NQ17

Column Letter: BU

Meaning: Did you have any co-workers die of COVID-19? If yes, how many? - Selected Choice

Data Type: Categorical

Acceptable Responses: 1, 2

1. Yes
2. No

## NQ17_1_TEXT

Column Letter: BV

Meaning: Did you have any co-workers die of COVID-19? If yes, how many? - Yes - Text

Data Type: Qualitative

Acceptable Responses: Responses are written.

## Q54

Column Letter: BW

Meaning: Please describe how your practice as a nurse or midwife has changed during the pandemic.

Data Type: Qualitative

Acceptable Responses: Responses are written.

## Q55

Column Letter: BX

Meaning: Please describe how your employer has responded to the pandemic. What did they do well? What did they not do well?

Data Type: Qualitative

Acceptable Responses: Responses are written.

## Q56

Column Letter: BY

Meaning: Please tell us about how the pandemic affected teamwork in your workplace. Please share about teamwork with fellow nurses or midwives, physicians, and other allied health professionals.

Data Type: Qualitative

Acceptable Responses: Responses are written.

## Q12

Column Letter: BZ

Meaning: How many years have you worked as a nurse or midwife?

Data Type: Categorical

Acceptable Responses: 1, 2, 3, 4, 5

1. 0-3 years
2. 4-6 years
3. 7-10 years
4. 11+ years
5. Prefer not to say

## NQ13

Column Letter: CA

Meaning: At what age do you plan to retire?

Data Type: Qualitative

Acceptable Responses: Responses are written.

## Q13

Column Letter: CB

Meaning: What population is the focus of your work? You may select more than one answer.

Data Type: Categorical

Acceptable Responses: 1, 2, 3, 4

1. Mothers and/or children under 5 years of age.
2. Children ages 6 to 18 years of age.
3. Adults between 19 and 64 years of age.
4. Older adults aged 65 years or more.

## NQ12

Column Letter: CC

Meaning: Which option best describes your role in nursing or midwifery?

Data Type: Categorical

Acceptable Responses: 1, 2, 3, 4

1. Practitioner or frontline worker
2. Educator
3. Management/Administrator
4. Other

## Q14

Column Letter: CD

Meaning: What is your area of clinical practice? Choose the closest match.- Selected Choice

Data Type: Categorical

Acceptable Responses: 1-10, 13-16

1. Intensive care unit/ward
2. Intermediate or step-down unit/ward
3. Emergency department
4. Medical surgical: please specify below.
5. General medicine
6. General Surgery
7. Labor & Delivery/Obstetrics/Maternal-Child Health
8. Psychiatric & mental health nursing
9. Operating Room/Theatre
10. Long term care, physical rehabilitation, or nursing home
13. Public Health/Community Health/Health Promotion
14. Other
15. Prefer not to say
16. Oncology

## Q14_4_TEXT

Column Letter: CE

Meaning: What is your area of clinical practice? Choose the closest match. - Medical-surgical combination: Please specify below. - Text

Data Type: Qualitative

Acceptable Responses: Responses are written.

## Q14_14_TEXT

Column Letter: CF

Meaning: What is your area of clinical practice? Choose the closest match. - Other - Text

Data Type: Qualitative

Acceptable Responses: Responses are written.

## Q14_Estonia – Estonia Respondents Only

Column Letter: CG

Meaning: What is your area of clinical practice? Choose the closest match.

Data Type: Categorical

Acceptable Responses: 1, 4-15

1. Primary health care
4. Labor & delivery/obstetrics/maternal-child health
5. Long-term care and nursing care in a care home
6. Outpatient clinic
7. Ambulance
8. Emergency medicine department
9. Intensive care
10. Operating room
11. Surgical care
12. Internal medicine department
13. Rehab
14. Other
15. Prefer not to say

## Q15

Column Letter: CH

Meaning: What nursing or midwifery degree do you hold?

Data Type: Categorical

Acceptable Responses: 1-5

1. Technical, enrolled, diploma, or associate degree
2. Bachelor’s
3. Master’s
4. Doctorate
5. Prefer not to respond

## Q16

Column Letter: CI

Meaning: Do you have a specialty certification?

Data Type: Categorical

Acceptable Responses: 1, 2

1. Yes
2. No

## Q17

Column Letter: CJ

Meaning: What type of organization do you work for? - Selected choice

Data Type: Categorical

Acceptable Responses: 1, 2, 3, 4, 5, 6

1. Primary Care
2. Ambulatory Care
3. Hospital
4. Home health care
5. Other
6. Prefer not to say

## Q17_6_TEXT

Column Letter: CK

Meaning: What type of organization do you work for? - Other - Text

Data Type: Qualitative

Acceptable Responses: Responses are written.

## Q18

Column Letter: CL

Meaning: Is the institution affiliated with a university or school of nursing?

Data Type: Categorical

Acceptable Responses: 1, 2, 3, 4

1. Yes
2. No
3. Not sure.
4. Prefer not to say

## Q19

Column Letter: CM

Meaning: Where is your place of work?

Data Type: Categorical

Acceptable Responses: 1, 2, 3, 4

1. Urban setting
2. Suburban setting
3. Rural setting
4. Prefer not to say

## NQ19

Column Letter: CN

Meaning: What kind of organization is your employer?

Data Type: Categorical

Acceptable Responses: 1, 2, 3, 4, 5, 6

1. Public
2. Not for Profit
3. Non-governmental organization
4. Private
5. Other
6. Prefer not to say

## NQ20

Column Letter: CO

Meaning: Are you a member of a labor union?

Data Type: Categorical

Acceptable Responses: 1, 2

1. Yes
2. No

## NQ6

Column Letter: CP

Meaning: What is your employment status?

Data Type: Categorical

Acceptable Responses: 1, 2, 3, 4, 5, 6

1. Full-time
2. Part-time (<24h/week)
3. Temporary contract
4. Per-diem
5. Other:

## NQ6_5_TEXT

Column Letter: CQ

Meaning: What is your employment status? - Other: - Text

Data Type: Qualitative

Acceptable Responses: Responses are written.

## NQ7

Column Letter: CR

Meaning: Since you are on a temporary or per-diem contract, is that:

Data Type: Categorical

Acceptable Responses: 1, 2, 3

1. By choice
2. Your only option
3. Other

## NQ7_3_TEXT

Column Letter: CS

Meaning: Since you are on a temporary or per-diem contract, is that: - Other: - Text

Data Type: Qualitative

Acceptable Responses: Responses are written.

## NQ8

Column Letter: CT

Meaning: Do you have more than one job in nursing or midwifery right now?

Data Type: Categorical

Acceptable Responses: 1, 2

1. Yes
2. No

## NQ9

Column Letter: CU

Meaning: Do you have another job outside of nursing or midwifery right now?

Data Type: Categorical

Acceptable Responses: 1, 2

1. Yes
2. No

## Q40

Column Letter: CV

Meaning: At any point between March 2020 and the present, were you furloughed or laid off from a nursing or midwifery job?

Data Type: Categorical

Acceptable Responses: 1, 2

1. Yes
2. No

## NQ14_1

Column Letter: CW

Meaning: When you were furloughed or laid off from your nursing or midwifery job, how long did it take you to: – Return to your original job

Data Type: Categorical

Acceptable Responses: 1, 2, 3, 4

1. 1 week to 3 months
2. 4-6 months
3. 7+ months
4. Not applicable

## NQ14_2

Column Letter: CX

Meaning: When you were furloughed or laid off from your nursing or midwifery job, how long did it take you to: – Find temporary employment as a nurse or midwife

Data Type: Categorical

Acceptable Responses: 1, 2, 3, 4

1. 1 week to 3 months
2. 4-6 months
3. 7+ months
4. Not Applicable

## NQ14_3

Column Letter: CY

Meaning: When you were furloughed or laid off from your nursing or midwifery job, how long did it take you to: – Find temporary employment outside of nursing or midwifery

Data Type: Categorical

Acceptable Responses: 1, 2, 3, 4

1. 1 week to 3 months
2. 4-6 months
3. 7+ months
4. Not Applicable

## NQ14_4

Column Letter: CZ

Meaning: When you were furloughed or laid off from your nursing or midwifery job, how long did it take you to: – Find part-time or full-time employment in nursing or midwifery

Data Type: Categorical

Acceptable Responses: 1, 2, 3

1. 1 week to 3 months
2. 4-6 months
3. 7+ months
4. Not applicable

## NQ3.1

Column Letter: DA

Meaning: Overall, I am satisfied with my current job.

Data Type: Categorical

Acceptable Responses: 1, 2, 3, 4, 5

1. Strongly Disagree
2. Disagree
3. Neither agree nor disagree
4. Agree
5. Strongly agree

## NQ3.2

Column Letter: DB

Meaning: Using your own definition of “burnout”, please choose one of the answers below.

Data Type: Categorical

Acceptable Responses: 1, 2, 3, 4, 5

1. I enjoy my work. I have no symptoms of burnout.
2. I am under stress, and don’t always have as much energy as I did, but I don’t feel burned out.
3. I am definitely burning out and have one or more symptoms of burnout, e.g. emotional exhaustion.
4. The symptoms of burnout that I’m experiencing won’t go away. I think about work frustrations a lot.
5. I feel completely burned out. I am at the point where I may need to seek help.

## NQ3.3

Column Letter: DC

Meaning: My professional values are well aligned with those of my department leaders.

Data Type: Categorical

Acceptable Responses: 1, 2, 3, 4, 5

1. Strongly Disagree
2. Disagree
3. Neither agree nor disagree
4. Agree
5. Strongly agree

## NQ3.4

Column Letter: DD

Meaning: The degree to which my care team works efficiently together is:

Data Type: Categorical

Acceptable Responses: 1, 2, 3, 4, 5

1. Excellent
2. Good
3. Satisfactory
4. Low/need improvement
5. Poor

## NQ3.5

Column Letter: DE

Meaning: I feel a great deal of stress because of my job

Data Type: Categorical

Acceptable Responses: 1, 2, 3, 4, 5

1. Strongly Disagree
2. Disagree
3. Neither agree nor disagree
4. Agree
5. Strongly agree

## NQ3.6

Column Letter: DF

Meaning: I stay after my shift is done to finish documentation (paper or electronic):

Data Type: Categorical

Acceptable Responses: 1, 2, 3, 4, 5, 6

1. Every time I work
2. 2-3 times/week
3. Once per week
4. Once every two weeks
5. Once a month
6. Very rarely

## NQ3.7

Column Letter: DG

Meaning: If I have to stay after my shift ends to finish documentation, I get paid overtime.

Data Type: Categorical

Acceptable Responses: 1, 2, 3

1. Yes
2. Sometimes
3. No

## NQ3.8

Column Letter: DH

Meaning: Which answer best describes the atmosphere in your primary work area?

Data Type: Categorical

Acceptable Responses: 1, 2, 3, 4, 5

1. Calm
2. Mostly quiet with periods of busyness
3. Busy, but reasonable
4. Constant activity, occasionally very intense
5. Hectic, chaotic

## NQ3.9

Column Letter: DI

Meaning: My control over my workload is:

Data Type: Categorical

Acceptable Responses: 1, 2, 3, 4, 5

1. Excellent
2. Good
3. Satisfactory
4. Low/need improvement
5. Poor

## NQ3.10

Column Letter: DJ

Meaning: Overall, my work environment is:

Data Type: Categorical

Acceptable Responses: 1, 2, 3, 4, 5

1. Excellent
2. Very Good
3. Good
4. Not too good
5. Bad

## NQ22.1

Column Letter: DK

Meaning: From your primary job, what is your monthly salary? Please respond in your country's currency?

Data Type: Qualitative

Acceptable Responses: Responses are written.

## Q216 – Thailand Respondents Only

Column Letter: DL

Meaning: From your primary job, what is your monthly salary?

Data Type: Categorical

Acceptable Responses: 1, 2, 3, 4, 5

1. 12,000-20,000 baht
2. 20,001-30,000 baht
3. 30,001-40,000 baht
4. 40,001-50,000 baht
5. >50,000 baht

## NQ22.2

Column Letter: DM

Meaning: Is your monthly salary enough to keep you out of poverty?

Data Type: Categorical

Acceptable Responses: 1, 2

1. Yes
2. No

## NQ22.3

Column Letter: DN

Meaning: Is your monthly salary enough to keep your household out of poverty?

Data Type: Categorical

Acceptable Responses: 1, 2

1. Yes
2. No

## NQ22.4

Column Letter: DO

Meaning: Are you the primary wage earner in your household?

Data Type: Categorical

Acceptable Responses: 1, 2

1. Yes
2. No

## NQ5.1

Column Letter: DP

Meaning: Are you thinking about leaving your current job in the next year?

Data Type: Categorical

Acceptable Responses: 1, 2, 3

1. Yes
2. No
3. Maybe

## NQ5.a

Column Letter: DQ

Meaning: Since you are thinking about leaving your current job in the next year, what would be your next career move?

Data Type: Categorical

Acceptable Responses: 1, 2, 3, 4, 5, 6

1. Take another job at my current employer but different practice setting
2. Take another job at the same kind of facility.
3. Take another job in a different care delivery context
4. Take another job outside of nursing or midwifery
5. Other
6. Prefer not to say

## NQ5.b

Column Letter: DR

Meaning: The career move may involve moving:

Data Type: Categorical

Acceptable Responses: 1, 2, 3, 4, 5, 6, 7, 9

1. From an urban to a suburban area
2. From an urban area to a rural area
3. From a suburban area to an urban area
4. From a rural area to a suburban area
5. From a rural area to an urban area
6. Not sure
7. Prefer not to say
9. From a suburban area to a rural area

## NQ5.c

Column Letter: DS

Meaning: Would your career move involve moving to another country?

Data Type: Categorical

Acceptable Responses: 1, 2, 3

1. Yes
2. No
3. Maybe

## NQ5.2

Column Letter: DT

Meaning: Are you thinking about leaving the nursing or midwifery profession in the next year?

Data Type: Categorical

Acceptable Responses: 1, 2, 3

1. Yes
2. No
3. Maybe

## Q30

Column Letter: DU

Meaning: Please list any education or training you received on the job to help you manage people infected with COVID-19 during the pandemic.

Data Type: Qualitative

Acceptable Responses: Responses are written.

## Q57

Column Letter: DV

Meaning: What nursing interventions or other treatments have you observed that improve outcomes for hospitalized people infected with COVID-19?

Data Type: Qualitative

Acceptable Responses: Responses are written.

## Q58

Column Letter: DW

Meaning: What nursing interventions or other treatments have you observed that DO NOT improve outcomes for hospitalized people infected with COVID-19?

Data Type: Qualitative

Acceptable Responses: Responses are written.

## Q33

Column Letter: DX

Meaning: Did you receive adequate training and education about the use of personal protective equipment?

Data Type: Categorical

Acceptable Responses: 1, 2, 3, 4

1. Yes
2. No
3. Not sure
4. Prefer not to say

## Q36

Column Letter: DY

Meaning: Did you receive any training about the spiritual, emotional, or psychological support needs of hospitalized people infected with COVID-19?

Data Type: Categorical

Acceptable Responses: 1, 2, 3, 4

1. Yes
2. No
3. Not sure
4. Prefer not to say

## NQ23_1

Column: DZ

Meaning: Please answer the following questions about the availability of resources to manage care for people infected with COVID-19.- Evidence-based medications to treat symptoms

Data Type: Categorical

Acceptable Responses: 1, 2, 3, 4, 5

1. Not available
2. Rarely available
3. Sometimes available
4. Usually available
5. Always available

## NQ23_2

Column: EA

Meaning: Please answer the following questions about the availability of resources to manage care for people infected with COVID-19.- Personal protective equipment

Data Type: Categorical

Acceptable Responses: 1, 2, 3, 4, 5

1. Not available
2. Rarely available
3. Sometimes available
4. Usually available
5. Always available

## NQ23_3

Column: EB

Meaning: Please answer the following questions about the availability of resources to manage care for people infected with COVID-19.- Intravenous infusion equipment

Data Type: Categorical

Acceptable Responses: 1, 2, 3, 4, 5

1. Not available
2. Rarely available
3. Sometimes available
4. Usually available
5. Always available

## NQ23_4

Column: EC

Meaning: Please answer the following questions about the availability of resources to manage care for people infected with COVID-19.- Oxygen and related infusion equipment

Data Type: Categorical

Acceptable Responses: 1, 2, 3, 4, 5

1. Not available
2. Rarely available
3. Sometimes available
4. Usually available
5. Always available

## NQ23_5

Column: ED

Meaning: Please answer the following questions about the availability of resources to manage care for people infected with COVID-19.- Mechanical ventilator

Data Type: Categorical

Acceptable Responses: 1, 2, 3, 4, 5

1. Not available
2. Rarely available
3. Sometimes available
4. Usually available
5. Always available

## NQ24

Column Letter: EE

Meaning: In the last month, how often have you had a patient infected with COVID-19 ask you to administer treatments that are not evidence-based?

Data Type: Categorical

Acceptable Responses: 1, 2, 3

1. Never
2. Daily
3. Weekly

## Q101

Column Letter: EF

Meaning: In the last month, how often have you had family members of patients infected with COVID-19 ask you to administer treatments that are not evidence-based?

Data Type: Categorical

Acceptable Responses: 1, 2, 3

1. Never
2. Daily
3. Weekly

## NQ25

Column Letter: EG

Meaning: In the last month, how often have you had a physician who has ordered you to administer treatments to people with COVID-19 that are not evidence-based?

Data Type: Categorical

Acceptable Responses: 1, 2, 3

1. Never
2. Daily
3. Weekly

## Q24

Column Letter: EH

Meaning: Have you been confirmed to have had a COVID-19 infection at any point between March 2020 and the present?

Data Type: Categorical

Acceptable Responses: 1, 2

1. Yes
2. No

## Q25

Column Letter: EI

Meaning: If you responded "yes" to experiencing symptoms of a coronavirus infection, please describe the symptoms you experienced.

Data Type: Qualitative

Acceptable Responses: Responses are written.

## Q28

Column Letter: EJ

Meaning: Did you have to work when you knew you were infected with the COVID-19 virus?

Data Type: Categorical

Acceptable Responses: 1, 2, 3

1. Yes
2. Yes, but only because my employer said I would lose my job
3. No

## NQ4.1

Column Letter: EK

Meaning: Did you receive a COVID-19 vaccination?

Data Type: Categorical

Acceptable Responses: 1, 2

1. Yes
2. No

## NQ4.4

Column Letter: EL

Meaning: IF you did not get a COVID-19 vaccination, please share why you did not.

Data Type: Qualitative

Acceptable Responses: Responses are written.

## NQ4.5

Column Letter: EM

Meaning: Since you did not get a COVID-19 vaccine, how often does your employer require you to get tested?

Data Type: Categorical

Acceptable Responses: 1, 2, 3, 4, 5

1. Weekly
2. Biweekly
3. Monthly
4. Not at all
5. Not currently employed

## NQ4.6

Column Letter: EN

Meaning: Where you live, what percentage of people do you think are vaccinated?

Data Type: Categorical

Acceptable Responses: 1, 2, 3, 4

1. 0-25%
2. 26-50%
3. 51-75%
4. 76-100%

## NQ4.7

Column Letter: EO

Meaning: Please rate your country's overall COVID-19 vaccination efforts.

Data Type: Categorical

Acceptable Responses: 1, 2, 3, 4, 5

1. Excellent
2. Very good
3. Good
4. Needs improvement
5. Poor

## Q35

Column Letter: EP

Meaning: Were you assigned to work in a different place in your organization (a.k.a redeployment) at any point between March 2020 and December 2023 as part of your organization’s pandemic response?

Data Type: Categorical

Acceptable Responses: 1, 2, 3, 4

1. Yes
2. No
3. Not sure
4. Prefer not to say

## NQ18

Column Letter: EQ

Meaning: If you were redeployed to a different work setting in your organization how long did you have to work there?

Data Type: Categorical

Acceptable Responses: 1, 3, 4, 5, 6, 7, 8, 9, 10, 11, 12

1. 1 week
3. 2 weeks
4. 3 weeks
5. 4 weeks
6. 5 weeks
7. 6 weeks
8. 7 weeks
9. 8 weeks
10. 9 weeks or more
11. Still redeployed
12. Prefer not to say

## Q38

Column Letter: ER

Meaning: Did you receive any extra pay or benefits because you were caring for people infected with COVID-19? - Selected Choice

Data Type: Categorical

Acceptable Responses: 1, 2, 3, 4

1. Yes. Please describe them below.
2. No
3. Not sure
4. Prefer not to say

## Q38_1_TEXT

Column Letter: ES

Meaning: Did you receive any extra pay or benefits because you were caring for people infected with COVID-19? - Yes. Please describe them below. - Text

Data Type: Qualitative

Acceptable Responses: Responses are written.

## Q39

Column Letter: ET

Meaning: Have you had any benefit reductions due to the pandemic, e.g. reductions in retirement contributions, changes in health insurance, etc.? - Selected Choice

Data Type: Categorical

Acceptable Responses: 1, 2

1. Yes. Please describe in the space below
2. No

## Q39_1_TEXT

Column Letter: EU

Meaning: Have you had any benefit reductions due to the pandemic, e.g. reductions in retirement contributions, changes in health insurance, etc.? - Selected Choice

Data Type: Qualitative

Acceptable Responses: Responses are written.

## Q41

Column Letter: EV

Meaning: Is COVID-19 considered as a workplace acquired infection by your employer?

Data Type: Categorical

Acceptable Responses: 1, 2, 3, 4

1. Yes
2. No
3. Not sure
4. Prefer not to say

## Q42

Column Letter: EW

Meaning: Have you been given the option to obtain alternate housing so you can protect your family from getting an infection?

Data Type: Categorical

Acceptable Responses: 1, 2, 3, 4

1. Yes
2. No
3. Not sure
4. Prefer not to say

## Q47

Column Letter: EX

Meaning: Right now, are you living in another place that is not your home to protect the people you live with from getting infected with COVID-19?

Data Type: Categorical

Acceptable Responses: 1, 2, 3

1. Yes
2. No
3. Prefer not to say

## Q44

Column Letter: EY

Meaning: Do you have to take more time to prepare for work when caring for people infected with COVID-19?

Data Type: Categorical

Acceptable Responses: 1, 2, 3, 4

1. Yes
2. No
3. Not sure
4. Prefer not to say

## Q45

Column Letter: EZ

Meaning: If you have to take more time to prepare for work when caring for people infected with COVID-19, how much longer on average?

Data Type: Categorical

Acceptable Responses: 1, 2, 3

1. Less than an hour
2. More than one hour but less than two hours
3. More than two hours

## Q105

Column Letter: FA

Meaning: Do you have to take more time to finish your work when caring for people infected with COVID-19?

Data Type: Categorical

Acceptable Responses: 1, 2, 3, 4

1. Yes
2. No
3. Not sure
4. Prefer not to say

## Q106

Column Letter: FB

Meaning: If you have to take more time to finish your work when caring for people infected with COVID-19, how much longer on average?

Data Type: Categorical

Acceptable Responses: 1, 2, 3

1. Less than an hour
2. More than one hour but less than two hours
3. More than two hours

## NQ8.1

*Column Letter:* FC

*Meaning:* What is one thing you wish had been done differently that would have made the response to the pandemic better and easier for you as a nurse or midwife?

*Data Type:* Qualitative

*Acceptable Responses:* Responses are written.

## Q60

Column Letter: FD

Meaning: Is there anything else you would like to add about living and working during the COVID-19 pandemic? Take as much space as you need.

Data Type: Qualitative

Acceptable Responses: Responses are written.
