# Supplementary material for: Assessing Pandemic Response Implementation and the Workforce Impact Among Nurses and Midwives Internationally: Protocol for an Exploratory, Pragmatic, Online Descriptive Study
Source: JMIR Res Protoc. 2026 Oct 27;15:e94179. doi: 10.2196/94179 (PMC13712254; doi:10.2196/94179)
Supplement: Supplementary file 3 [file resprot-v15-e94179-s003.docx]

**Round 1 Consortium Countries**

Bangladesh

Costa Rica

Ghana

Hong Kong (China)

Israel

Mexico

Mongolia

Poland

Saudi Arabia

Slovenia

South Korea

Ukraine

United States

**Round 2 Consortium Countries (All Round 1 Countries Continued Participation in Round 2)**

Albania

Antigua and Barbuda

Argentina

Bangladesh

Bolivia

Brazil

Brunei

Cambodia

Cameroon

Chile

Colombia

Costa Rica

Cote D'Ivoire

Democratic Republic of Congo

Dominican Republic

Ecuador

Egypt

El Salvador

Estonia

Ethiopia

Finland

France

Georgia

Germany

Ghana

Greece

Grenada

Guatemala

Guyana

Honduras

Hong Kong/China

India

Indonesia

Italy

Iraq (Kurdistan)

Jamaica

Japan

Kenya

Khazakhstan

Kosovo

Latvia

Lebanon

Lithuania

Malawi

Malta

Mexico

Mongolia

Montenegro

Nigeria

Pakistan

Panama

Paraguay

Peru

Philippines

Poland

Qatar

Rwanda

Saudi Arabia

Singapore

Slovenia

Somaliland/Somalia

South Africa

South Korea

Spain

St. Lucia

Switzerland

Tanzania

Thailand

Trinidad & Tobago

Turkey

Uganda

United Arab Emirates

Uruguay

Venezuela

Zambia

Zimbabwe
